# Am80-lipid nanoparticles serve as an enteric mucosal adjuvant following parenteral immunization with inactivated polio vaccine

**DOI:** 10.1126/sciadv.aea5433

**Published:** 2026-06-03

**Authors:** Behnaz Eshaghi, Erika Yan Wang, Sevinj Mursalova, Timothy A. Forster, Ninaad Lasrado, Jinbi Tian, Dorin Artzi, Stacey Qiaohui Lin, Zongting Wu, Xin Yang, Wontaek Chung, Ilin Sadeghi, Johnny Garcia, Ziqi Chen, Olivia Sheridan, Rishi Das, Alicia Lau, Aki Gurram, Bernardo A. Mainou, Kathryn A. V. Jones, Yi Liu, Dan H. Barouch, Ulrich von Andrian, Robert Langer, Ana Jaklenec

**Affiliations:** ^1^ David H. Koch Institute for Integrative Cancer Research, Massachusetts Institute of Technology, Cambridge, MA 02139, USA.; ^2^ Center for Virology and Vaccine Research, Beth Israel Deaconess Medical Center, Harvard Medical School, Boston, MA 02215, USA.; ^3^ Department of Electrical Engineering, Massachusetts Institute of Technology, Cambridge, MA 02139, USA.; ^4^ The Division of Comparative Medicine, Massachusetts Institute of Technology, Cambridge, MA 02139, USA.; ^5^ Department of Immunology, Harvard Medical School, Boston, MA 02115, USA.; ^6^ The Ragon Institute of MGH, Massachusetts Institute of Technology and Harvard, Cambridge, MA 02139, USA.; ^7^ Division of Viral Diseases, National Center for Immunization and Respiratory Diseases, Centers for Disease Control and Prevention, 1600 Clifton Rd NE, Atlanta, GA 30329, USA.; ^8^ Regenerative Medicine Program, Ottawa Hospital Research Institute, Ottawa, ON K1H8L6, Canada.; ^9^ Department of Cellular and Molecular Medicine, Faculty of Medicine, University of Ottawa, Ottawa, ON K1H 8M5, Canada.; ^10^ Department of Chemical Engineering, Massachusetts Institute of Technology, Cambridge, MA 02139, USA.

## Abstract

Enteric pathogens are a major contributor to the global disease burden, necessitating vaccines capable of inducing robust gastrointestinal mucosal immunity. Achieving this response is especially challenging with inactivated or subunit vaccines, which lack the ability of live-attenuated formulations to mimic the natural infection process needed to induce strong intestinal mucosal immunity. Specifically, the inactivated polio vaccine (IPV), administered parenterally, elicits strong systemic immunity but fails to induce the mucosal IgA responses required to fully block poliovirus transmission, a critical step toward complete eradication. To address this limitation, we developed a clinically translatable and scalable nanoparticle-based intestinal mucosal adjuvant by encapsulating Am80, a small hydrophobic molecule known to promote intestinal mucosal immunity, within nanoparticles (NPs) designed for lymph node delivery, without requiring antigen modification, encapsulation, or adsorption. Encapsulation in NPs eliminated the need for the use of organic solvents, reduced toxicity, and prevented degradation, while lipid nanoparticles (Am80-LNPs) were engineered for sustained release over 3 to 5 days with high encapsulation efficiency (78 ± 9%). Cy5-labeled Am80-LNPs localized to draining lymph nodes within 6 hours and were retained for up to 72 hours. Coadministration of Am80-LNPs with the licensed IPV-2 in a Wistar rat model significantly boosted IPV-2–specific fecal IgA by 20-fold compared to IPV-2 alone and threefold over free Am80. These findings demonstrate that Am80-LNPs significantly enhance IPV-2–specific mucosal immunity in vivo and suggest their potential as a potent mucosal adjuvant against other enteric pathogens.

## Teaser

Vitamin A derivative Am80–loaded lipid nanoparticles boost intestinal mucosal immunity for injectable inactivated polio vaccines.

## INTRODUCTION

Enteric pathogens, such as *Vibrio cholerae*, *Salmonella typhi*, enterotoxigenic *Escherichia coli* (ETEC), rotavirus, and poliovirus, are major causes of gastrointestinal infections worldwide, contributing substantially to global morbidity and mortality (*1*–*4*). Cholera alone affects an estimated 1.3 to 4 million people and causes 28,000 to 143,000 deaths annually (*5*), while typhoid fever results in over 9 million cases and 110,000 deaths each year (*6*), and ETEC is responsible for up to 280 million cases and more than 50,000 deaths annually (*7*, *8*). Effective vaccination strategies against these pathogens require the induction of mucosal immunity in the gastrointestinal tract, where these infections often originate (*8*, *9*). However, achieving robust mucosal responses in the gastrointestinal tract is challenging, especially with inactivated or subunit vaccines that lack the ability of live-attenuated formulations to mimic the natural infection process to induce intestinal mucosal immunity (*10*, *11*). Several adjuvants, substances added to vaccines to enhance immune responses, show promise in generating gastrointestinal immunity but they also present substantial challenges (*12*). For instance cholera toxin (*13*) and heat-labile enterotoxin (LT) (*14*) are associated with toxicity, while others, such as mucoadhesive polymer nanoparticles (NP) (*15*, *16*) (e.g., chitosan) or CpG oligodeoxynucleotides (*17*) and flagellin (*18*, *19*) often require specific administration routes like oral, nasal, or pulmonary delivery, limiting their applicability to parenteral vaccines.

This limitation is especially relevant in the case of polio vaccination. Currently, two classes of vaccines are available against polio: the orally administered live-attenuated oral polio vaccine (OPV) and the parenterally administrated inactivated polio vaccine (IPV) (*20*). While OPV is cost-effective and easy to administer, it carries significant risks, including vaccine-associated paralytic poliomyelitis and potential reversion to a neurovirulent form of poliovirus, which can result in strains with transmissibility and virulence similar to wild polioviruses (*20*). In addition, poliovirus replication for 28 years has been observed in a vaccinated immunodeficient individual, highlighting how immune compromise can permit prolonged viral shedding and evolution even in vaccinated hosts (*21*). Therefore, continuous use of OPV presents an ongoing challenge to achieving complete polio eradication. While IPV addresses the major drawbacks of OPV and produces immunoglobulin G (IgG) antibodies to protect against the dissemination of poliovirus (systemic immunity) throughout the body, it fails to induce mucosal IgA responses in the intestinal tissues (*20*, *22*, *23*), which are the primary sites of infection. This lack of mucosal immunity leaves the intestinal mucosa of vaccinated individuals vulnerable, enabling transmission and posing a critical gap in eradication efforts (*24*). Since the global initiative to eradicate polio began in 1988, wild poliovirus type 1 (WPV1) continues to be endemic in Afghanistan and Pakistan, with 12 WPV1 cases reported in 2023. In addition, circulating vaccine-derived poliovirus (cVDPV) continues to pose a substantial challenge, causing 524 cases across 32 countries in 2023 (*25*). Therefore, it is crucial to establish a vaccination strategy that integrates the safety and systemic immunogenicity of IPV with the mucosal immunity offered by OPV.

Previous studies have demonstrated that repeated injections of all-trans-retinoic acid (atRA) can promote intestinal mucosal immunity by inducing small intestine imprinting, a phenomenon involving programming of T and B lymphocytes to acquire gut-homing properties, enabling them to migrate specifically to the small intestine and initiate localized intestinal mucosal immune responses (*26*, *27*). During antigen exposure, atRA has been shown to enhance the ability of effector T and B cells in the draining lymph nodes (LNs) to home to the small intestine by upregulating the expression of gut-homing molecules α4β7 and CCR9, and to aid in the class-switching to generate IgA producing plasma cells (*28*–*31*). While previous studies with retinoid-based mucosal adjuvants have demonstrated mucosal imprinting, clinical translation has been limited by complex NP structures, multicomponent fabrication, lyophilization requirements, and insufficient characterization of key parameters such as release kinetics and encapsulation efficiency (*32*, *33*). With the broadening therapeutic applications of retinoids, various synthetic analogs have been developed with improved properties. For instance, Am80, a synthetic retinoid that is approximately 10 times more potent, less toxic, and more chemically stable than atRA (*34*, *35*), was developed that could serve as a more effective molecule to induce mucosal immunity. Administration and delivery of free retinoid compounds pose several challenges such as (i) toxicity and local inflammation or rash at the injection site (*36*), which limits tolerability (ii) poor aqueous solubility (*37*) necessitating high doses or the use of organic solvents such as dimethyl sulfoxide (DMSO) or ethanol, as well as in emulsifiers such as PEG400, which can lead to significant tissue toxicity at the injection sites, increasing the risk of adverse effects, (iii) chemical instability, short half-life, and low bioavailability (*38*–*40*), leading to rapid degradation and reduced therapeutic efficacy, which restricts therapeutic potential, and (iv) the requirement for multiple daily injections, complicating their clinical translation as mucosal adjuvant (*32*, *33*).

To address these challenges, we developed an intestinal mucosal adjuvant by encapsulating Am80, a small hydrophobic molecule, within a NP. We selected an NP carrier for Am80 as recent studies have demonstrated that NPs within the size range of ~100 nm can be effectively trafficked to LNs, where they can initiate stronger immune responses (*33*, *41*, *42*). To select the most suitable material for encapsulating Am80, we initially evaluated several materials (table S1). NPs of approximately 100 nm exhibit optimal lymphatic transport and LN retention (*33*, *41*, *42*). Therefore, we excluded sub–100 nm carriers (β-cyclodextrin or Pluronic F127) from further evaluation. Subsequent characterization focused on NPs with hydrodynamic diameters of 150 to 180 nm [poly(lactic-*co*-glycolic acid) (PLGA), PLGA–poly(ethylene glycol) (PEG), acetalated dextran, and LNPs]. Although several nanocarriers produced detectable responses in vivo, only Am80-LNPs elicited reproducible, robust, and pronounced antigen-specific IgA, consistent with Am80 sustained release over approximately 5 days, which matches the window required for small-intestine imprinting. In addition, only Am80-LNPs were retained in LNs for up to 72 hours. Thus, we engineered Am80-LNPs to effectively overcome the identified challenges. Specifically, by encapsulating Am80 in LNPs, we reduced toxicity by minimizing direct contact and accumulation of the free compound at the injection site, thereby lowering the risk of local inflammation and tissue irritation. Encapsulation also resolved the issue of poor aqueous solubility, enabling stable suspensions of Am80-LNPs in phosphate-buffered saline (PBS) for easy administration without the need for toxic organic solvents such as DMSO or ethanol. Furthermore, the LNP matrix provided a protective environment, addressing the chemical instability, short half-life, and low bioavailability of free Am80 by shielding the molecule from rapid degradation. The engineered Am80-LNPs allowed sustained release of Am80 over several days, eliminating the need for daily injections and enhancing therapeutic efficacy. Consistent with sustained release, using ex vivo IVIS imaging, we demonstrated retention of Cy5-Am80-LNPs in draining LNs (dLNs) for up to 72 hours. We show sustained release of Am80 from LNPs induced intestinal mucosal immunity by small intestine imprinting.

To test the translational potential of our Am80-LNP formulation, we evaluated its potency as enteric mucosal adjuvants in Wistar rats immunized with Sabin IPV (sIPV) serotype 2 (IPV-2). We also included Am80-encapsualted PEGylated lipid-coated PLGA NPs (Am80-PLGA NPs) as a comparison to the Am-80-LNP group. Unlike Am80-LNPs, Am80-PLGA NPs exhibit a burst release, with 90% of Am80 being released within a single day. This comparison further underscores the critical role of Am80 release kinetics in modulating mucosal immune responses. Am80-LNPs produced 20-fold higher IPV-2–specific fecal IgA than IPV-2 alone, threefold higher than free Am80, and fourfold higher than Am80-PLGA NPs. This improved response in Am80-LNPs is attributed to the sustained release kinetic of Am80 in Am80-LNPs (80% released over 5 days) compared to burst release in Am80-PLGA NPs (90% within a day). Our results further demonstrate that encapsulation of Am80 in NPs alone is insufficient for inducing robust mucosal immunity; appropriate release kinetics are essential, as evidenced by the fecal IgA responses. Neutralizing and IgG titers confirmed significant systemic immunity and provided immune correlation to protection against polio. Small intestine IgA staining showed a twofold increase in the Am80-LNP group, and cytokine analysis revealed statistically significant higher levels of interferon-γ (IFN-γ), tumor necrosis factor–α (TNF-α), interleukin-2 (IL-2), IL-4, IL-6, IL-10, and IL-17A compared to other groups. In addition, LNPs are biodegradable, biocompatible, and Food and Drug Administration–approved, which further facilitate the use of Am80-LNPs as an intestinal mucosal adjuvant (*43*). These findings underscore the use of Am80-LNPs as a potentially powerful enteric mucosal adjuvant for parenterally administered vaccines.

## RESULTS

### Fabrication and characterization of Am80-encapsulated NPs

**Fig. 1.**
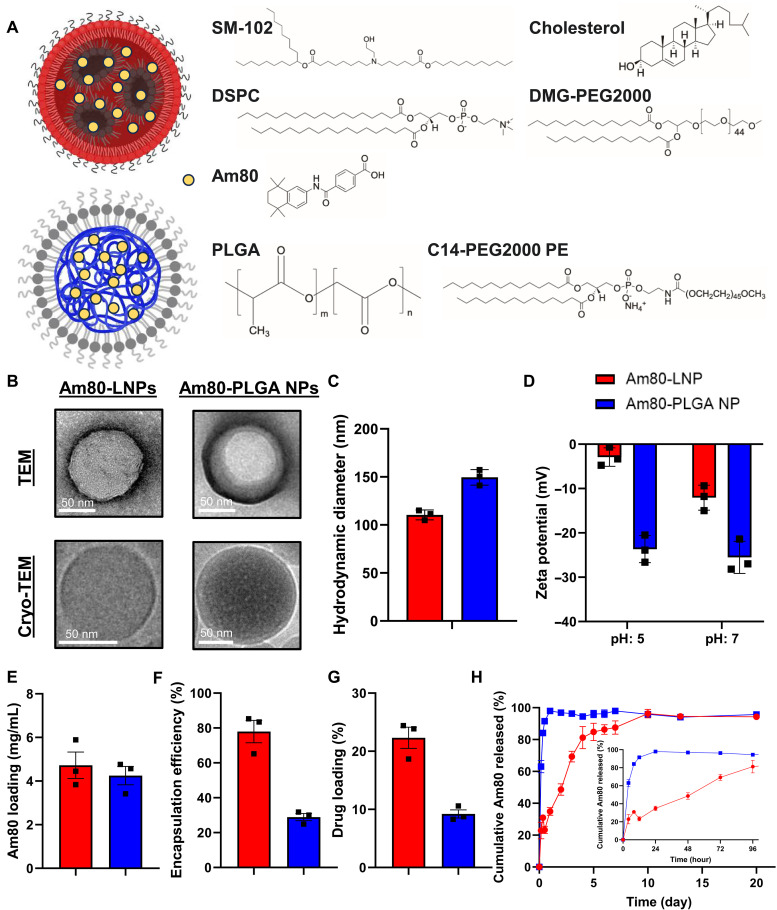
Structure, composition, and characterization of Am80-NPs. (**A**) Schematic presentation of Am80-loaded LNPs and PLGA NPs. Chemical structure of the lipids (SM-102, DSPC, cholesterol, and DMG-PEG2000) in Am80-LNPs, PLGA and C14 PEG2000 PE in Am80-PLGA NPs, and Am80 molecule are shown. (**B**) High-resolution TEM and cryo-TEM images of Am80-LNPs and Am80-PLGA NPs. Scale bar, 50 nm. (**C** and **D**) Hydrodynamic diameter and zeta potential of Am80-LNPs and Am80-PLGA NPs. Error bars represent SD of three replicates. (**E** to **G**) Am80 loading (E), encapsulation efficiency (F), and drug loading (G) in Am80-LNPs and Am80-PLGA NPs. Error bars represent standard deviation of three replicates. (**H**) Release kinetics of Am80-NPs at 37°C in 1× PBS over a duration of 21 days. The cumulative percentage of Am80 released is plotted. Error bars represent SD of three replicates. The inset shows the zoomed-in view of days 1 to 4.

To establish a new and potent mucosal adjuvant for IPV, we encapsulated Am80 within LNPs (Am80-LNPs). Am80-loaded PEGylated lipid-coated PLGA NPs (Am80-PLGA NPs) were included as a comparison to Am80-LNPs (*43*, *44*). Am80-LNPs and Am80-PLGA NPs were synthesized through nanoprecipitation (*45*, *46*). The composition of Am80-LNPs is as follows: 50 mol % SM-102, 38.5 mol % cholesterol, 10 mol % DSPC, and 1.5 mol % DMG-PEG2000. The core material of Am80-PLGA NPs is PLGA with an intrinsic viscosity of 0.32 to 0.44 dl g^−1^ and a molecular weight (MW) of 24,000 to 38,000 g mol^−1^ and C14PEG2000PE is used as the lipid membrane (*46*). The formation of a lipid layer around the polymeric NP core is driven by hydrophobic interactions between the lipid tails and the polymer core (*47*). The schematic representation of the structure and composition of Am80-loaded LNPs and PLGA NPs is shown in Fig. 1A. High-resolution transmission electron microscopy (TEM) images, negatively stained with sodium phosphotungstate Na_3_P(W_3_O_10_)_4_ to enhance contrast by using electron-dense material, allowing visualization of their overall shape, size, and morphology, and cryogenic-TEM (cryo-TEM) images of Am80-LNPs and Am80-PLGA NPs are depicted in Fig. 1B. Additional TEM and cryo-TEM images of larger fields of view of Am80-NPs are included in fig. S1. The TEM and cryo-TEM images reveal the spherical morphology and well-preserved structural integrity of Am80-LNPs and Am80-PLGA NPs following fabrication and purification. The hydrodynamic diameter, as determined by dynamic light scattering (DLS), of Am80-LNPs and Am80-PLGA NPs are 110 ± 4 nm and 150 ± 7 nm, respectively. The 100 to 150 nm size range indicates optimal size for efficient cellular uptake (*41*, *43*, *46*) The zeta potentials of the Am80-LNPs at pH 5 and 7 are −3 ± 2 mV and −12 ± 2 mV and for Am80-PLGA NPs at pH 5 and 7 are −24 ± 2 mV and −25 ± 3 mV. The less negative zeta potential of Am80-LNPs at pH 5 compared to pH 7 reflects the expected protonation of ionizable lipids under acidic conditions (*43*). In contrast, the consistent zeta potential observed for Am80-PLGA NPs across pH 5 and 7 aligns well with the nonionizable nature of PEGylated-PLGA NPs (*46*). The overall surface charge of −10 to −30 mV at pH 7 (physiological pH) is within the range (≈ −30 to +30 mV) compatible with efficient cellular uptake (*41*). The hydrodynamic diameters and zeta potentials of all the investigated NPs are summarized in Fig. 1 (C and D).

To measure Am80 loaded into NPs, the final NP products were dissolved in acetonitrile, and Am80 concentration was then determined by high-performance liquid chromatography (HPLC). The average concentration of Am80 loaded in LNPs and PLGA NPs are 4.7 ± 0.8 and 4.2 ± 0.6 mg/ml, respectively (Fig. 1E). These values correspond to encapsulation efficiencies of 78 ± 9% for Am80-LNPs and 29 ± 3% for Am80-PLGA NPs (Fig. 1F) and drug loading are 22 ± 3% and 9 ± 1% for Am80-LNPs and Am80-PLGA NPs, respectively (Fig. 1G). This reflects a 2.7-fold higher encapsulation efficiency and a 2.4-fold increase in drug loading for Am80-LNPs compared to Am80-PLGA NPs.

Next, we measured the release kinetics of Am80 from LNPs and PLGA NPs at 37°C in 1× PBS over 20 days (Fig. 1H). Am80-LNPs demonstrated controlled release, with 80% of Am80 being released over 5 days, in contrast to the faster burst release from Am80-PLGA NPs, where 90% was released within 1 day. These findings highlight the potential of Am80-LNPs as a promising mucosal vaccine adjuvant, as controlled release of Am80 enables sustained access of Am80 by immune cells in the dLNs, which is needed for successful small intestine imprinting. This aligns with previous studies using atRA-encapsulated vehicles, which demonstrated the importance of controlled release in enhancing immune activation (*32*, *33*). While prior RA-based systems have demonstrated efficacy (*32*, *33*), Am80-LNPs offer improved aqueous stability without lyophilization or dual-particle architectures, enabling a more commercially scalable and translational platform for mucosal vaccination. Throughout the release experiment (over 21 days), the size of the NPs was consistently monitored using DLS (fig. S2). The consistent hydrodynamic diameter of NPs in the range of 100 to 150 nm indicates excellent stability of investigated NPs. Furthermore, to monitor the storage and stability of Am80-NPs at 4°C, final products were kept at 4°C and the size, surface charge, and drug loading was monitored for 28 days (fig. S3). Cell viability assays using 3-(4,5-dimethylthiazol-2-yl)-2,5-diphenyltetrazolium bromide (MTT) demonstrated no cytotoxicity associated with Am80-NPs in HeLa cells (fig. S4).

Overall, we successfully encapsulated Am80 into LNPs and PEGylated PLGA NPs, resulting in stable NPs with consistent size and morphology (in the range of 100 to 150 nm, an optimal range for cellular uptake and efficient delivery), maintaining high stability at 4°C over an extended period of time, without any cell toxicity. Given the sustained release kinetics of Am80 observed in LNPs (80% cargo released over 5 days) compared to the burst release from PLGA NPs (90% released within a day) in vitro, we hypothesized that an extended availability of Am80 by LNPs would enhance adjuvanticity and favor immune cells for successful small intestine imprinting and enhanced gut mucosal immune responses, but these needed further testing in vivo.

**Fig. 2.**
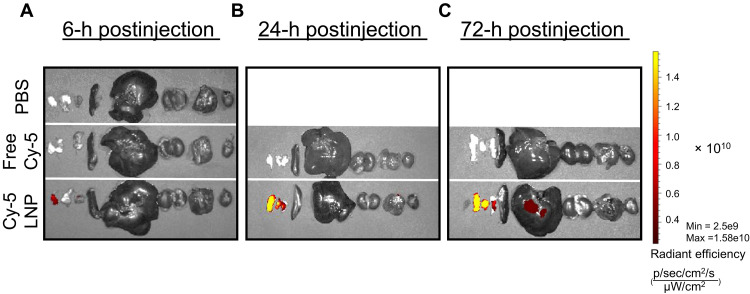
Biodistribution of Cy5–Am80-LNPs following subcutaneous administration in Wistar rats. (**A** to **C**) Ex vivo IVIS imaging of major organs at 6 (A), 24 (B), and 72 hours (h) (C) after subcutaneous injection of free Cy5 or Cy5–Am80-LNPs. PBS-injected rats were included and imaged at 6 hours as a background control. Organs are shown left to right as: LNs, spleen, liver, kidneys, lungs, and heart. For LNs, samples were harvested and presented in the order inguinal, popliteal, and iliac. The same radiant-efficiency scale was applied to all images.

We next assessed the ex vivo biodistribution of Cy5-labeled Am80-LNPs in Wistar rats (Fig. 2). Fluorescence signal localized to the inguinal dLNs 6 hours postinjections and, by 24 hours, was also evident in the popliteal and iliac dLNs. Signal in the inguinal, popliteal, and iliac dLNs persisted through 72 hours, aligning with the time window required for small intestine imprinting. A low-level liver signal appeared at 72 hours, consistent with normal hepatic clearance of NPs. No detectable signal was observed in spleen, kidney, lung, or heart. Additional animals and independent replicates are presented in fig. S5. Together, these data support sustained dLN retention with minimal off-target accumulation and reinforce the translatability of Am80-LNPs.

### Am80-LNPs induce robust intestinal mucosal immunity determined by production of IPV-2–specific fecal IgA

**Fig. 3.**
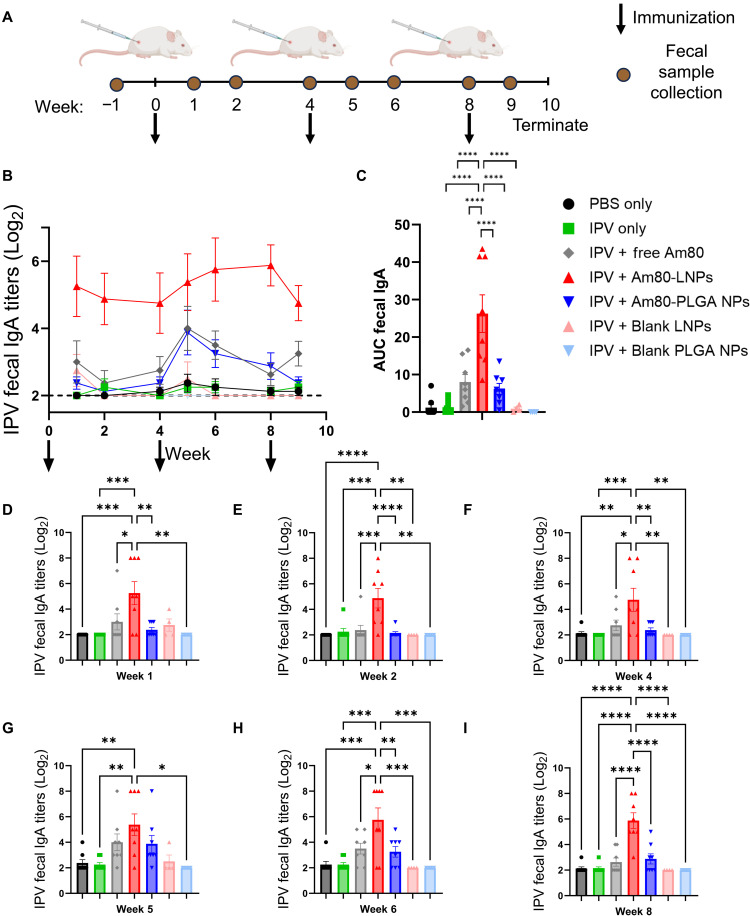
Am80-LNPs induce mucosal immunity as measured by IPV-2–specific fecal IgA. (**A**) Schematic overview of the experimental design. Wistar rats were subcutaneously immunized at weeks 0, 4, and 8 with 12.5 DU of sIPV serotype 2 and 1.8 mg of Am80, either in free form or encapsulated in LNPs or PLGA NPs. Free Am80 was administered on days 1 to 3 at a dose of 0.6 mg per day. Blank LNPs and PLGA NPs were included as controls. Number of rats: *n* = 8 for all groups, except *n* = 4 for blank LNPs and PLGA NPs. Arrows indicate immunization, and the brown circle represents the frequency of fecal sample collection. (**B**) IPV-2–specific IgA titers in fecal samples throughout the study, arrows indicate the immunization. The dashed line indicates a log2 value of 2, representing the lower limit of detection. (**C**) Area under the curve (AUC) analysis of the IPV-2–specific IgA titers presented in (B). (**D** to **I**) IPV-2–specific IgA titers for individual animals at different time points are shown. Error bars represent SEM. Statistical *P* values were determined using one-way ANOVA followed by a Tukey post hoc test. Significance levels are indicated as *P ≤ 0.05, **P ≤ 0.01, ***P ≤ 0.001, and ****P ≤ 0.0001. Nonsignificant comparisons are not shown in the figure.

Mucosal immunity can be measured via production of IgA (*20*, *22*, *48*). To evaluate the induction of mucosal immunity following the coadministration of Am80-NPs and IPV-2, Wistar rats were immunized subcutaneously at weeks 0, 4, and 8 with IPV serotype 2 and Am80-NPs. Fecal samples of all the animals were collected before immunization at week 0 and used in the assays as the baseline. The IPV-2 dose was 12.5 DU and the Am80 dose was 1.8 mg. To align with previous studies on atRA, for the free Am80 compound group, the 1.8 mg dose was delivered through three consecutive injections on days 1, 2, and 3, each consisting of 0.6 mg (*32*, *33*). Sham groups receiving only PBS injections and a group that received IPV-2 only, without any Am80, were included in our study. To distinguish the effects of Am80 from the immunostimulatory effects of NPs, we also included groups that were given blank LNPs and PLGA NPs. Size and zeta potential measurements of blank LNPs and PLGA NPs were summarized in fig. S6. The absorbance of NPs at wavelengths of 370 to 390 nm was used to determine the dose of blank NPs to match the dose of Am80-NPs (fig. S7). The schematic in Fig. 3A summarizes the fecal sample collection and vaccination schedule. We measured IPV-2–specific IgA titers in fecal samples as a key indicator of a mucosal immune response (Fig. 3B). Our results demonstrate that administration of IPV-2 with Am80-LNPs induced IPV-2–specific IgA responses after the initial prime vaccination, suggesting a rapid onset of intestinal mucosal immunity. After the week-4 injections, the groups receiving free Am80 and Am80-PLGA NPs also induced detectable IPV-2–specific fecal IgA. Blank LNPs and PLGA NPs did not induce detectable IPV-2–specific IgA titers in fecal samples, confirming the specific role of Am80 in inducing intestinal mucosal vaccine response. Notably, the IPV-2–specific IgA titers in the Am80-LNP group remained consistently high throughout the entire study, indicating a sustained immune response. Further analysis of the area under the curve (AUC) (*49*) throughout the study demonstrated that the Am80-LNP group showed the highest total IPV-specific fecal IgA production, which was statistically significant compared to the other groups, with levels 20-fold higher than rats vaccinated with IPV-2 only, threefold higher than rats vaccinated with IPV-2 and free Am80 injections, and fourfold higher than rats vaccinated with IPV-2 and Am80-PLGA NP (Fig. 3C). Overall, AUC analysis demonstrated the superior efficacy of Am80-LNPs coadministered with IPV-2 in inducing a strong and sustained intestinal mucosal immune response. Detailed individual animal responses for each week are presented in Fig. 3 (D to I) and fig. S8, highlighting the consistency and robustness of the intestinal mucosal immune response induced by Am80-LNP and IPV-2 vaccinated rats. This early and strong immune response observed in Am80-LNP and IPV-2-vaccinated animals indicated a rapid generation of small intestine-tropic vaccine response which may be attributed to the slower release kinetics of Am80 observed in Am80-LNPs compared to Am80-PLGA NPs (Fig. 1H). In a second cohort monitored to 20 weeks, fecal IgA remained elevated above baseline throughout (fig. S9), indicating durable intestinal mucosal responses of Am80-LNPs. While previous RA-based NP systems provided valuable insights into mucosal vaccine delivery (*32*, *33*), fecal IgA responses were either not a primary focus or assessed at limited time points. In contrast, our longitudinal analysis reveals a rapid and sustained induction of IPV-specific fecal IgA beginning as early as 2 weeks postvaccination, highlighting the potent mucosal adjuvanticity of our platform.

### Systemic immune responses remain uncompromised by coadministration of Am80-LNPs with vaccine

**Fig. 4.**
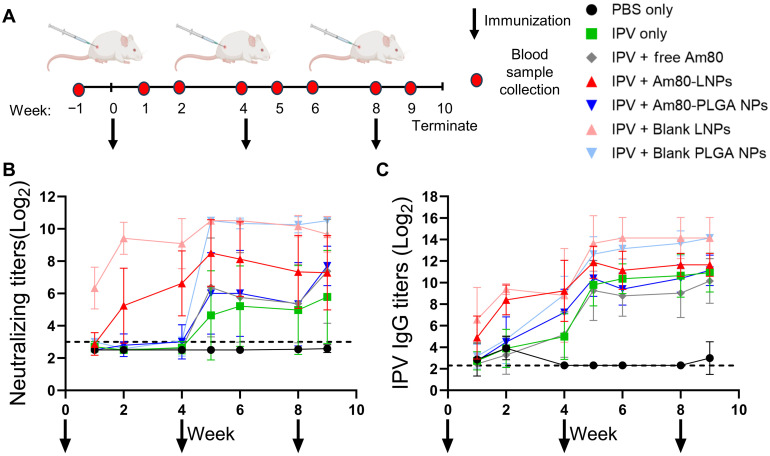
Systemic immune responses are improved by the coadministration of Am80-LNPs and the vaccine. (**A**) Schematic overview of the experimental design. Wistar rats were subcutaneously immunized at weeks 0, 4, and 8 with 12.5 DU of sIPV serotype 2 and 1.8 mg of Am80, either in free form or encapsulated in LNPs or PLGA NPs. Free Am80 was administered on days 1 to 3 at a dose of 0.6 mg per day. Blank LNPs and PLGA NPs were included as controls. Number of rats: *n* = 8 for all groups, except *n* = 4 for blank LNPs and PLGA NPs. Arrows indicate immunization, and the red circle represents the frequency of blood sample collection. (**B**) Neutralizing titers in serum samples throughout the study, arrows indicate the immunization. The dashed line indicates a log2 value of 3, representing seropositivity and protection against polio. (**C**) IgG titers in serum samples throughout the study, arrows indicate the immunization. The dashed line indicates a log2 value of 2.1, representing the lower limit of detection.

Figure 4A shows the blood sample collection and vaccination schedule. To assess systemic immune responses and protection against polio, we measured serum neutralizing antibody (NAb) titers and IPV-2–specific IgG titers against poliovirus serotype 2 (*50*). The serum NAb titers are presented in Fig. 4B. Log2 values higher than 3 show seropositivity (*51*), which is the immune correlate of protection against polio in animals. Both Am80-LNP and blank LNP groups showed protective poliovirus NAb responses after the prime injections, with poliovirus NAb detected in all groups following the week-4 injections. No detectable poliovirus NAb were observed in the PBS sham groups. The NAb assay results demonstrate titers above the seroprotective threshold, with animals receiving Am80, either in free form or encapsulated in NPs, as well as those receiving empty NPs, suggesting robust adjuvanticity of Am80 and NPs resulting in induction of protective immune responses against polio virus that is at least comparable to, or higher than, that of the IPV-2–only group. Individual animal responses per week, including naïve rat serum collected before vaccination (week −1), are shown in fig. S10.

We next measured IPV-2–specific IgG titers in serum samples using enzyme-linked immunosorbent assay (ELISA; Fig. 4C). Serum samples of all the animals before immunization at week −1 are used as the baseline. Similar to the NAb titers, early production of detectable IPV-2–specific IgG was observed in rats vaccinated with Am80-LNPs and blank LNPs following the prime immunization, indicating a pronounced adjuvant-like effect of combining IPV-2 vaccine with LNPs. Titers in all other experimental groups (except PBS) also increased gradually after the prime injections, reaching their highest levels after the week 4 injections. All IgG titers plateaued by week 5. Individual animal responses per week are shown in fig. S11.

Similar to the poliovirus NAb titers, the results for IPV-2–specific IgG titers confirm that animals receiving Am80 demonstrated a systemic immune response against polio that is at least comparable to, or greater than, the antigen-specific IgG responses observed in the IPV-2–only group. Overall, these results confirm that coadministration of Am80 with IPV-2 does not impair the systemic immune response and effectively induces a strong immune response against polio, and that all rats vaccinated with IPV-2 and Am80, either as the free compound or encapsulated in NPs, demonstrated protective NAb titers above the seroprotective threshold and indicated robust systemic immune responses through IgG titers. In the 20-week follow-up cohort, serum IgG and Nab titers remained above baseline (fig. S9), consistent with a durable systemic immunity. This robust systemic response, coupled with sustained mucosal immunity, is achieved using a World Health Organization–prequalified IPV formulation, highlighting direct clinical translational potential compared to previously studied RA-based strategies using only model antigens such as *Chlamydia muridarum* major outer membrane protein and ovalbumin (*32*, *33*).

### Enhancement of IgA production in the small intestine of rats immunized with Am80-LNPs

**Fig. 5.**
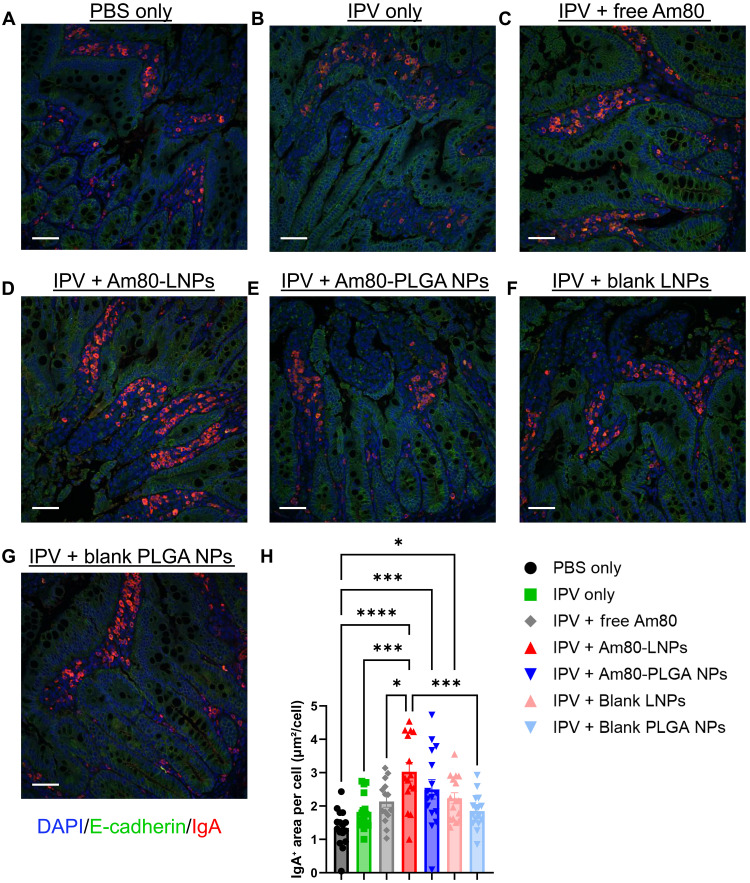
Increased IgA production in the small intestine of rats immunized with Am80-LNPs. (**A** to **G**) Confocal *z*-stack representative images of small intestine tissues of different groups stained for IgA. Blue indicates nuclei of the cells, green indicates epithelial cells, and red indicates IgA. Scale bar, 50 μm. (**H**) IgA-positive area within the intestinal sections, normalized to the number of cells. Error bars represent standard error of the mean (SEM). A total of 16 different fields of view from *n* = 4 animals, with 4 different tissue sections per animal are included in the analysis. Statistical *P* values were determined using one-way ANOVA followed by a Tukey post hoc test. Significance levels are indicated as *P ≤ 0.05, **P ≤ 0.01, ***P ≤ 0.001, and ****P ≤ 0.0001. Nonsignificant comparisons are not shown in the figure.

To evaluate the local intestinal mucosal immune response, we performed IgA immunohistochemistry (IHC) staining on the small intestine of Wistar rats harvested at week 10, the terminal time point of the study, following immunization at weeks 0, 4, and 8. This analysis was conducted to assess IgA production in the intestinal mucosa (*52*, *53*), a key indicator of mucosal immunity. Immediately after harvesting, 5-mm segments of the distal ileum (10 cm from the ileocecal junction) were fixed and processed. The processed tissue sections were then subjected to IHC staining using an anti-IgA antibody, where E-cadherin was used to stain epithelial cells and 4′,6-diamidino-2-phenylindole (DAPI) was used to stain the nuclei of all cells (*54*). Confocal *z*-stack representative images from each experimental group are presented in Fig. 5 (A to G), where red indicates IgA, green indicates epithelial cells, and blue indicates cells. Next, we quantified the IgA-positive area within the intestinal sections (Fig. 5H). The average IgA-positive area was significantly higher in the group immunized with Am80-LNPs and IPV-2 compared to all other groups, averaging two times higher than both the IPV-2–only and free Am80 groups, indicating enhanced mucosal immunity. This highlights the role of Am80-LNPs in augmenting local intestinal mucosal immune responses. Additional confocal images of rat intestines treated with Am80-LNPs and IPV-2 are presented in fig. S12. This enhanced IgA production in the Am80-LNP group further reinforces the superior ability of Am80-LNPs to promote IgA production compared to other formulations or conditions such as free Am80.

### Enhanced serum cytokine and chemokine responses following mucosal adjuvantation with Am80-LNPs

**Fig. 6.**
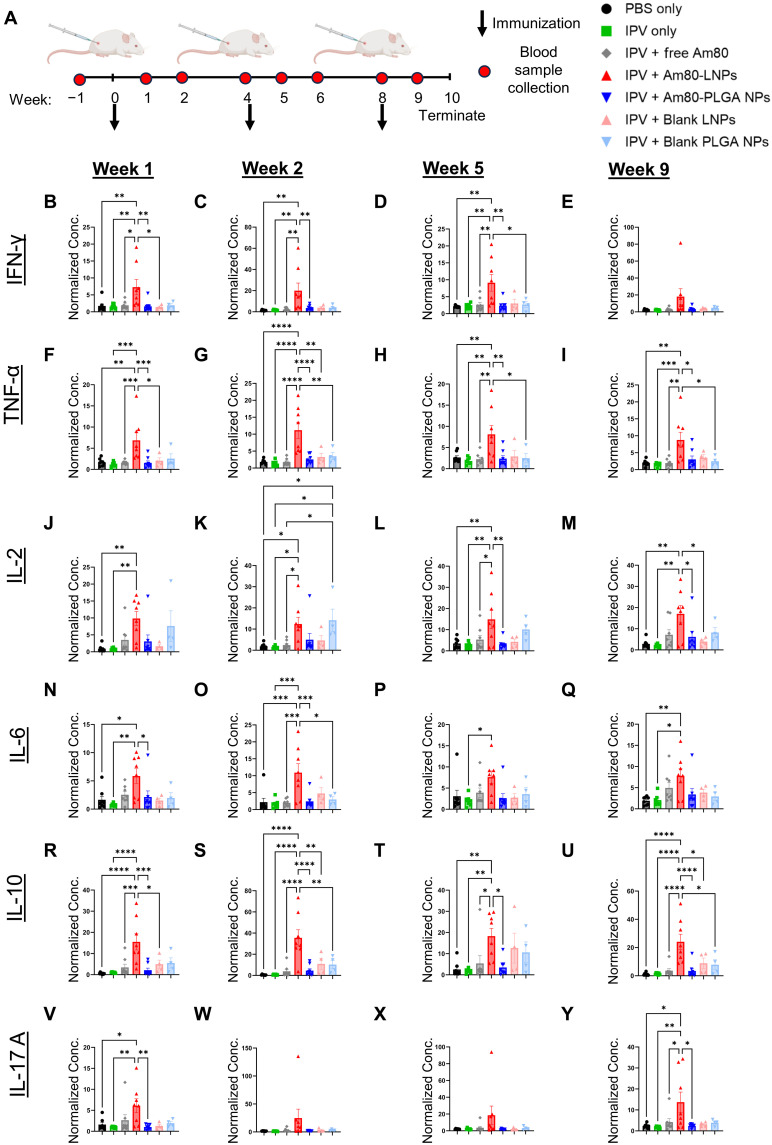
Elevated cytokine levels in serum samples from rats immunized with Am80-LNPs. (**A**) Schematic overview of the experimental design. Wistar rats were subcutaneously immunized at weeks 0, 4, and 8 with 12.5 DU of sIPV serotype 2 and 1.8 mg of Am80, either in free form or encapsulated in LNPs or PLGA NPs. Free Am80 was administered on days 1 to 3 at a dose of 0.6 mg per day. Blank LNPs and PLGA NPs were included as controls. Number of rats: *n* = 8 for all groups, except *n* = 4 for blank LNPs and PLGA NPs Arrows indicate immunization, and the red circle represents the frequency of blood sample collection. (**B** to **E**) IFN-γ, (**F** to **I**) TNF-α, (**J** to **M**) IL-2, (**N** to **Q**) IL-6, (**R** to **U**) IL-10, and (**V** to **Y**) IL-17A levels at week 1, 2, 5, and 9. All data are normalized to the baseline serum levels of each naïve rat. Error bars represent SEM. Statistical *P* values were determined using one-way ANOVA followed by a Tukey post hoc test. Significance levels are indicated as *P ≤ 0.05, **P ≤ 0.01, ***P ≤ 0.001, and ****P ≤ 0.0001. Nonsignificant comparisons are not shown in the figure.

To further evaluate the potential of Am80-LNPs as an enteric mucosal adjuvant for vaccine development, we assessed the ensuing immune response by monitoring cytokine and chemokine production in serum at peak time points postimmunizations (*55*). Proinflammatory type 1 cytokines such as IFN-γ and IL-12 are essential for cell-mediated immunity and protection against intracellular pathogens, whereas type 2 cytokines, including IL-4, IL-5, and IL-13, are preferentially associated with humoral immunity (*56*, *57*). To evaluate these responses, including other relevant proinflammatory (IL-2, IL-1β, TNF-α, IL-6, and IL-17A) and anti-inflammatory (IL-10) cytokines in our vaccinated animal cohorts, we used the Luminex xMAP assay for multiplexed quantification of 27 rat cytokines, chemokines, and growth factors (*58*). Undiluted sera collected at peak response time points postimmunizations (weeks 1, 2, 5, and 9) were analyzed (fig. S13), with baseline sera obtained from naïve rats at week −1 (before immunization). Individual animal responses at weeks 1, 2, 5, and 9 are shown in figs. S14 to S17, respectively. The detected concentration ranges for each biomarker are depicted in fig. S18. Our analysis revealed that adjuvantation of Am80-LNPs with IPV-2 induced robust levels of serum cytokine responses such as IFN-γ, TNF-α, IL-2, IL-4, IL-6, IL-10, and IL-17A, which were consistently higher than other groups across peak immune response time points postimmunizations (Fig. 6 and figs. S13 to S17). These results suggest engagement of multiple effector modalities of both the innate and adaptive immune system to mount critical antiviral immune responses.

**Fig. 7.**
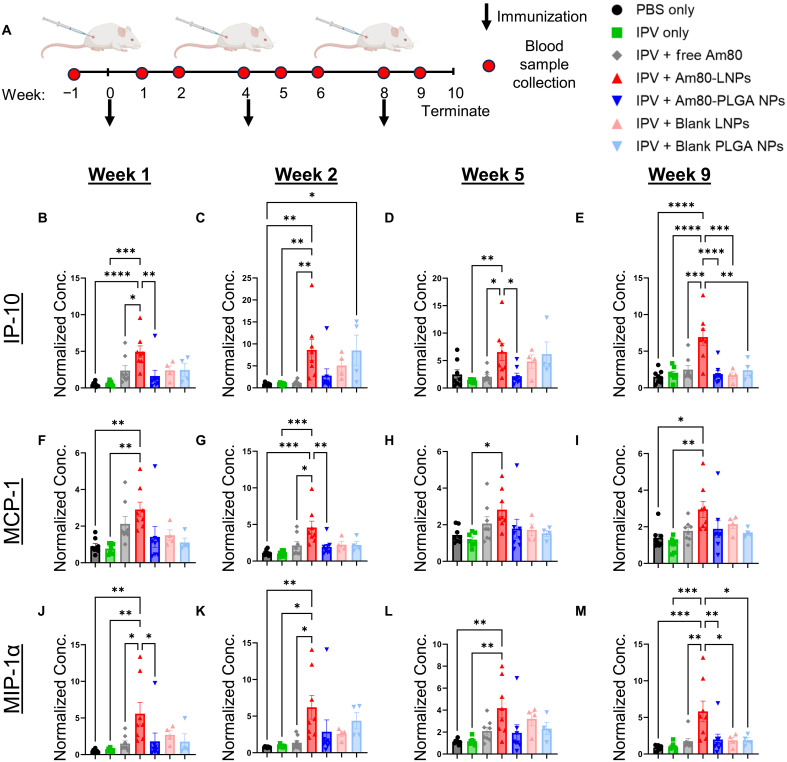
Elevated chemokine levels in serum samples from rats immunized with Am80-LNPs at peak-immunity indicate enhanced antiviral immune responses. (**A**) Schematic overview of the experimental design. Wistar rats were subcutaneously immunized at weeks 0, 4, and 8 with 12.5 DU of sIPV serotype 2 and 1.8 mg of Am80, either in free form or encapsulated in LNPs or PLGA NPs. Free Am80 was administered on days 1 to 3 at a dose of 0.6 mg per day. Blank LNPs and PLGA NPs were included as controls. Number of rats: *n* = 8 for all groups, except *n* = 4 for blank LNPs and PLGA NPs. Arrows indicate immunization, and the red circle represents the frequency of blood sample collection. (**B** to **E**) IP-10, (**F** to **I**) MCP-1, and (**J** to **M**) MIP-1α levels at week 1, 2, 5, and 9. All data are normalized to the baseline serum levels of each naïve rat. Error bars represent SEM. Statistical *P* values were determined using one-way ANOVA followed by a Tukey post hoc test. Significance levels are indicated as *P ≤ 0.05, **P ≤ 0.01, ***P ≤ 0.001, and ****P ≤ 0.0001. Nonsignificant comparisons are not shown in the figure.

We next evaluated chemokine responses in sera from vaccinated rats (Fig. 7), including IP-10 (IFN-γ–induced protein 10), MCP-1 (monocyte chemoattractant protein-1/CCL2), and MIP-1α (macrophage inflammatory protein-1α). These chemokines play significant roles in immune cell recruitment and activation, resulting in antiviral immune responses. Our data showed elevated levels of IP-10 at peak immunity in rats immunized with Am80-LNPs and IPV-2, suggesting enhanced recruitment of T cells and NK cells, contributing to the antiviral and proinflammatory response. MCP-1 levels were also increased in this group, indicating active monocyte recruitment. In addition, MIP-1α levels were elevated in rats vaccinated with IPV-2 and Am80-LNPs, highlighting its role in macrophage and other immune cell recruitment during early immune activation. These findings collectively demonstrate a robust chemokine-driven antiviral immune response by Am80-LNPs. This consistent, yet transient, enhanced cytokine and chemokine production at peak immunity time points after repeated vaccinations further validates the role of Am80-LNPs as potent adjuvants in promoting robust antiviral immune responses, compared to other vaccine regimens.

To further assess cellular immune activation induced by Am80-based formulations, we performed intracellular cytokine staining and ELISPOT analyses on splenocytes collected from vaccinated animals at week 10 (fig. S19). Notably, animals immunized with IPV-2 adjuvanted with Am80-LNPs exhibited increased frequencies of IFN-γ–producing CD4^+^ and CD8^+^ T cells compared to IPV alone, free Am80, and Am80-PLGA NP groups. Consistent with these findings, ELISPOT analysis demonstrated elevated numbers of IFN-γ–secreting cells in the Am80-LNP group, indicating enhanced antigen-specific cellular responses.

## DISCUSSION

Developing effective vaccines against enteric pathogens, which play a major role in the global health burden, requires the induction of a strong mucosal immune response within the gastrointestinal tract (*8*, *9*, *12*). However, generating and maintaining such responses remains difficult, particularly with inactivated or subunit vaccines that lack the ability of live-attenuated vaccines to mimic the natural infections to induce the intestinal mucosal immunity (*10*). This limitation is particularly evident with the IPV, which is administered parenterally and generates strong systemic immunity, but fails to induce mucosal IgA responses in the gastrointestinal tract, an essential component for fully blocking poliovirus transmission and ultimately eradicating polio worldwide (*20*, *22*, *23*). To address this need, we developed Am80-encapsulated NPs as an enteric mucosal adjuvant following parenteral immunization with IPV-2. Mechanistically, previous studies have shown that retinoic acid and its derivatives (Am80 and atRA) promote small-intestine imprinting and enhance intestinal mucosal immunity by upregulating the gut-homing receptors α4β7 and CCR9 on T and B cells and by facilitating IgA class switching to generate IgA-producing plasma cells (*26*–*31*). As a comparison to Am80-LNPs, we also included Am80-loaded PEGylated lipid-coated PLGA NPs (Am80-PLGA NPs). To evaluate the induction of intestinal mucosal immunity, we measured IPV-specific fecal IgA in a polio rat model after coadministration of IPV and Am80-LNPs following parenteral immunization (Fig. 3B). Am80-LNP group had the statistically significant highest overall fecal IgA levels, 20 times higher than rats vaccinated with IPV alone, three times higher than those receiving IPV with free Am80 multiple repeated injections, and four times higher than those vaccinated with IPV and Am80-PLGA NPs. Our release studies suggest that the sustained and controlled release kinetics of Am80 from LNPs (80% in 5 days) compared to the burst release of Am80 from PLGA NPs (90% within a day) (Fig. 1H), have prolonged the exposure of the immune system to the adjuvant and thereby enhanced the mucosal immune responses by small intestine imprinting (*59*). Furthermore, we demonstrated the retention of Am80-LNPs in the dLNs up to 72 hours (Fig. 2). This finding underscores the importance of Am80-LNPs as an enteric mucosal adjuvant and highlights the specificity of the immune response elicited by Am80-LNPs. The rapid onset of robust mucosal immunity, detectable after the initial vaccination dose, could potentially be advantageous for outbreak scenarios or resource-limited settings where multiple vaccine doses may be challenging. To decouple the effects of Am80 from the immunostimulatory properties of the NPs, we included blank LNPs and PLGA NPs. The groups that received blank LNPs and PLGA NPs did not produce significant IgA in fecal samples (Fig. 3B), confirming that the mucosal immune response was specifically driven by Am80 in LNPs and not by the NP carriers alone. The neutralizing and IgG antibody titers presented in Fig. 4 confirm that Am80-LNPs do not compromise the systemic immune response and effectively induce protection against polio. In addition, the production of neutralizing and IgG antibodies in the blank LNP and PLGA NP groups aligns with the previous findings suggesting that NPs have an immunostimulatory effect on systemic immune responses (*60*). Consistent with this, single-cell analyses of NP adjuvants have shown that NPs can activate innate inflammatory pathways and promote germinal center B cell differentiation, even without mRNA or DNA payloads, highlighting that NPs by themselves can act as immunostimulatory agents and modulate systemic antibody responses (*61*–*63*). We also monitored a second cohort up to 20 weeks to assess durability of immune response. Fecal IgA, serum IgG, and NAb titers remained above baseline throughout this period (fig. S9), confirming sustained mucosal and systemic responses. In addition, the average IgA-positive area within the intestinal sections (Fig. 5H) was significantly and approximately two times higher in the group immunized with Am80-LNPs compared to all other groups. These findings are consistent with the elevated IPV-specific fecal IgA levels observed in ELISA assays (Fig. 3), further supporting the efficacy of Am80-LNPs as a potent enteric mucosal adjuvant. The robust IgA production suggests that Am80-LNPs not only enhance the overall immune response but also specifically target intestinal mucosal surfaces, providing localized protection. This is particularly important in the context of enteric mucosal pathogens, where a strong mucosal immune response in intestine is crucial for preventing infection and subsequent transmission (*64*). Last, we explored the potential of Am80-LNPs as an enteric mucosal adjuvant to induce robust antiviral immune responses by examining circulating cytokine and chemokine production at peak immunity after repeated vaccinations (*55*) (Figs. 6 and 7 and figs. S13 to S17). The consistent, yet transient, elevation of antiviral cytokine levels in the Am80-LNP group compared to others at peak immunity suggests that Am80-LNPs induce an early and balanced protective antiviral immune response, critical for protection against enteric viruses (*56*). In addition our data showed increased IL-17 cytokine responses in sera and IPV-specific IgA levels in the intestine of Am80-LNP–vaccinated rats compared to other groups, indicating a potential role for IL-17–producing cells in enhancing protective mucosal immunity (*65*). However, future experiments are needed to further elucidate how Am80-LNPs act on different immune and stromal cell types and their potential contributions to enhancing localized and systemic antiviral immunity.

In conclusion, our findings in a rat model demonstrate the induction of mucosal immunity after coadministration of Am80-LNPs and IPV-2 and highlight the importance of developing vaccination strategies that effectively target mucosal enteric sites (*12*). This is particularly critical for polio, as the virus has yet to be fully eradicated. Without a robust enteric mucosal adjuvant, IPV struggles to prevent viral replication and transmission in the gut, which is essential for stopping the spread of poliovirus (*23*). The use of IPV, a licensed human vaccine used globally, underscores the translational relevance and impact of our findings. Unlike prior model-antigen studies, our Am80-LNP adjuvant demonstrates direct clinical potential, enhancing injectable vaccine efficacy by promoting robust mucosal imprinting. Developing enteric mucosal adjuvants, such as Am80-LNPs, could play a pivotal role in achieving complete global eradication of polio by allowing the generation of mucosal immune responses with IPVs that do not lead to the seeding of new vaccine-associated infections. To date, there is no commercial adjuvant approved for coadministration with IPV to enhance mucosal immunity. A recent phase 1 randomized study evaluating the use of dmLT as an adjuvant with IPV showed no significant differences in fecal IgA levels or viral shedding between the IPV alone and IPV + dmLT groups (*66*). The addition of an enteric mucosal adjuvant, such as Am80-LNPs, could potentially address the current gap in achieving robust mucosal immunity, which is essential for complete eradication of poliovirus by preventing transmission at the gut level. By inducing both systemic and mucosal immune responses, this approach may offer a strategy for enhancing the efficacy of IPV and similar parenteral vaccines (*67*). Although our study highlights the potential of Am80-LNPs in inducing mucosal immunity in a preclinical rat model, future investigations in human subjects will be essential to confirm their translational applicability.

The use of Am80-LNPs as a mucosal adjuvant represents an important advancement in the vaccine area as the controlled release of Am80 from LNPs provides sustained exposure that effectively achieves small intestine imprinting to induce mucosal immune responses. This approach not only addresses the need for enhanced enteric mucosal immunity but also provides a foundation for the development of vaccines that could better control the spread of infectious diseases (*68*). Future studies will focus on validating the efficacy of Am80-LNPs using a challenge model, assessing long-term mucosal immune responses, and exploring their application to other enteric pathogens beyond poliovirus. To the best of our knowledge, this is the first report to encapsulate Am80 in LNPs to serve as an enteric mucosal adjuvant following parenteral immunizations. This work could potentially be applied to other vaccines targeting enteric pathogens such as *V. cholerae*, *S. typhi*, ETEC, and rotavirus, where enhanced and durable intestinal mucosal immunity is desired. The ability of Am80-LNPs to induce robust and long-lasting immune responses suggests that this strategy could be pivotal in advancing vaccine design, providing more effective protection against a wide range of mucosal pathogens. As we continue to face global health challenges, this approach may play a crucial role in the development of next-generation vaccines, offering a previously unexplored pathway to achieving comprehensive and durable immunity.

## MATERIALS AND METHODS

### Fabrication of Am80-loaded NPs

To prepare the Am80-LNPs, Am80 (stock: 50 mg/ml in ethanol) was mixed with lipids in ethanol at a molar ratio of 50:10:38.5:1.5 (SM-102:DSPC:cholesterol:DMG-PEG2000; lipid stock 10 mg/ml in ethanol), maintaining an Am80 to lipids ratio of 1:2.5 (wt/wt). For fluorescent formulations (Cy5–Am80-LNPs), 1,2-dioleoyl-*sn*-glycero-3-phosphocholine-*N*-(cyanine 5) was added to the ethanol lipid mixture at a tracer molar fraction to enable in vivo imaging system (IVIS) imaging. The solution was then rapidly added to citrate buffer (at pH 4) in a volume ratio of 3:1 (aqueous:ethanol), followed by 1 min of vortex mixing with pulses. Am80-PLGA NPs were fabricated using a one-step nanoprecipitation procedure as described previously. PLGA (20 mg/ml in acetonitrile), Am80 (50 mg/ml in ethanol), and C14-PEG2000 PE (10 mg/ml in ethanol) were mixed together at a 1:2 weight ratio Am80:PLGA, using a lipid/polymer weight ratio of 15%. The organic phase was then added to deionized water, and the final mixture was sonicated in a bath sonicator (Branson 5510, Branson Ultrasonics, Danbury, CT) for 5 min. The Am80-LNPs or Am80-PLGA NPs were purified by three washing cycles (4000*g*, 15 min) using an Amicon Ultra centrifugal filter (MilliporeSigma, Burlington, MA) with a MW cutoff of 10 kDa to remove the organic solvent and free drug and lipid molecules. The purified Am80-LNPs or Am80-PLGA NPs were then dispersed in 1× PBS. The same procedure was followed to make blank LNPs and PLGA NPs, in the absence of Am80. PLGA [poly(d,l-lactide-*co*-glycolide); lactide:glycolide, 50:50; ester terminated; intrinsic viscosity, 0.32 to 0.44 dl g^−1^; and MW, 24,000 to 38,000 (RG503)], acetonitrile, and ethanol were purchased from Sigma-Aldrich (St. Louis, MO). Lipids were purchased from Avanti Polar Lipids (Alabaster, AL). Am80 (S4260) was purchased from Selleckchem (Houston, TX).

### TEM and Cryo-TEM imaging

The carbon-coated copper TEM grids were prepared by adding Am80-LNPs and Am80-PLGA NPs, followed by blotting to remove any excess solution. For TEM imaging, the samples were stained with a 1% aqueous solution of phosphotungstic acid, after which the excess stain was removed, and the samples were dried at room temperature (RT) before imaging. For cryo-TEM imaging, samples were rapidly plunge-frozen using a 930 Gatan Cryoplunge 3. Imaging for all samples was performed using a JEOL 2100 FEG microscope with an acceleration voltage of 200 kV.

### DLS and zeta-potential measurements

The size and zeta potential were measured using a Zetasizer Nano ZS ZEN3600 (Malvern, Worcestershire, UK). To determine the NP size, the NPs were diluted with Milli-Q water. A 10 mM NaCl solution at pH 5 and 7 was used to assess the zeta potential of the NPs.

### Quantification of Am80 in NPs using HPLC

To quantify the amount of Am80 encapsulated in LNPs and PLGA NPs, the NPs were dissolved in acetonitrile, releasing the encapsulated drug. The free drug was then analyzed using a Waters HPLC (e2695) system equipped with a Syncronis C18 column (no. 97105-154630, Thermo Fisher Scientific, Waltham, MA) featuring a 5 μm particle size, 4.6 mm internal diameter, and 150 mm length. The mobile phase comprised an 85:15 (v/v) mixture of methanol and 150 mM sodium acetate (Sigma-Aldrich, St. Louis, MO) in water. The analysis was performed in isocratic mode with a flow rate of 1 ml/min over a total runtime of 10 min. Am80 was detected at a wavelength of 286 nm, with an average retention time of 4 min. Calibration curves were generated by running calibration solutions alongside the samples in each HPLC run. A stock solution of Am80 (10 mg/ml) was prepared in acetonitrile, and calibration solutions were created by diluting the stock solution with acetonitrile to obtain concentrations of 0.5, 1, 5, 10, and 100 μg/ml. The encapsulation efficiency and drug loading percentages were calculated using the following formulas

$$\text{Encapsulation efficiency}\;\left(\%\right)=\frac{\text{weight of}\;\text{Am}80\;\text{in}\;\text{NPs}}{\text{weight of total}\;\text{Am}80\;\text{input}}\times 100$$

$$\;\text{Drug loading}\;\left(\%\right)=\frac{\text{weight of}\;\text{Am}80\;\text{in}\;\text{NPs}}{\text{weight of total}\;\left(\text{NPs}+\text{Am}80\right)}\times 100$$

### Am80 release kinetics determination

To assess the drug release profile, Am80-LNPs or Am80-PLGA NPs were placed into Slide-A-Lyzer MINI dialysis microtubes with a 3500 Da MW cutoff (Thermo Fisher Scientific, Waltham, MA). The microtubes were dialyzed in 4 liters of 1× PBS buffer at 37°C. The PBS buffer was refreshed every 24 hours for the first 10 days of the dialysis process and then every 2 days until day 20. At each designated time point, the NP solution from three individual microtubes was collected, and each sample was mixed with acetonitrile to dissolve the NPs. The released Am80 was then quantified via HPLC analysis, as previously described. Throughout the release studies, the size of Am80-LNPs and Am80-PLGA NPs was monitored using DLS measurements.

### Am80-NP characterization during storage at 4C over 4 weeks

The size and zeta potential of Am80-LNPs and Am80-PLGA NPs stored at 4°C were measured weekly over a 4-week period, as previously described. At each time point, drug loading in the NPs stored at 4°C was determined by washing the NPs (14,000*g*, 3 min) using an Amicon 0.5-ml ultracentrifugal filter (MilliporeSigma, Burlington, MA) with a 10 kDa MW cutoff to potentially remove any free drug that may have leaked from the NPs during storage.

### Cell viability MTT assays

HeLa cells were seeded in a 96-well plate at a concentration of 1 × 10^4^ cells per well. The cells were then treated with Am80-LNPs and Am80-PLGA NPs, each containing 150 nM of Am80, for 1, 6, and 24 hours. At each time point, the cells were washed with 1× PBS and incubated with RPMI medium containing 10% MTT solution (5 mg/ml) for 3 hours at 37°C. Following the removal of the MTT solution, a DMSO and ethanol mixture (1:1 ratio) was added and mixed thoroughly. Absorbance was measured at 570 and 650 (reference) nm using a TECAN Infinite M PLEX (Tecan Trading AG, Switzerland).

### Blank NPs dose determination

To match the dose of blank NPs to that of Am80-NPs, the absorbance of both Am80-NPs and blank NPs was measured at 370, 375, 380, 385, and 390 nm. The dose of Am80-NPs was determined by HPLC analysis. The absorbance values of the blank NPs were then normalized to those of the Am80-NPs.

### Animal experiments

Wistar rats (6 to 8 weeks old) were purchased from Charles River Laboratories and housed in an MIT animal facility. All the following procedures were performed in accordance with an animal protocol approved by the Massachusetts Institute of Technology Institutional Animal Care and Use Committee (IACUC) (protocol number 2304000509).

### Rat immunization and blood and fecal sample collection and processing

Rats were anesthetized using a chamber with 2.5% isoflurane in oxygen throughout our studies for injections and bleeds. Wistar rats were immunized subcutaneously at weeks 0, 4, and 8 with IPV serotype 2 (Takeda, Cambridge, MA) (12.5 DU) and Am80 (1.8 mg). The study included several groups: a sham group receiving only PBS, an IPV-2–only group, an IPV-2 with free Am80 group, and groups receiving Am80-LNPs or Am80-PLGA NPs. In addition, control groups were treated with blank LNPs or PLGA NPs. For the free Am80 group, the 1.8 mg group dose was divided into three consecutive daily injections of 0.6 mg each, administered on days 1 (the same day as IPV-2 immunization), 2, and 3 (1 and 2 days following the IPV-2 immunization day, respectively).

Blood samples were collected via tail bleed using a 24 gauge, ¾ inch catheter (no. 533440, McKesson, Richmond, VA) at week −1 (naïve) and at weeks 1, 2, 4, 5, 6, 8, and 9. The serum was separated using serum gel microtubes (no. 50809211, Thermo Fisher Scientific, Waltham, MA) and centrifuged at 10,000*g* for 3 min. The serum samples were aliquoted and stored at −20°C until further analysis.

Fresh feces were collected by placing each rat into a separate cage, and 1 g (equivalent to approximately seven to nine large pellets) was collected from each animal. Fecal samples were placed on ice immediately upon collection until further handling. Following collection, half of the samples were used for immediate processing, while the remaining samples were stored at −20°C. For fresh processing, fecal pellets underwent breakdown and resuspension using 1× protease inhibitor (cOmplete, EDTA-free Protease Inhibitor Cocktail, no. 11873580001, Sigma-Aldrich, St. Louis, MO) in PBS at 200 mg/ml. Samples were subsequently transferred to omni tubes (no. 19-624, OMNI INTERNATIONAL INC, Kennesaw, GA) without beads (after discarding the beads) and homogenized for two cycles and each cycle 1 min (at 4 m/s) using an OMNI Bead Mill Homogenizer (model: Bead Ruptor Elite, no. 19-042E, OMNI INTERNATIONAL INC, Kennesaw, GA). The samples were incubated for 10 min on ice between the two cycles. After homogenization, the samples were centrifuged at 10,000*g* for 10 min at 4°C and then the supernatant was collected and aliquoted and stored at −20°C until further analysis.

### Biodistribution of Cy5–Am80-LNPs and ex vivo IVIS imaging

Cy5–Am80-LNPs were prepared as described above. Female Wistar rats were injected subcutaneously with Cy5–Am80-LNPs, free Cy5, or PBS (vehicle control) at the same Am80 or dye-equivalent dose used in immunization studies. At 6, 24, or 72 hours postinjection, animals were euthanized, and major organs including inguinal, popliteal, and iliac dLNs, liver, spleen, kidneys, lungs, and heart were collected for ex vivo fluorescence imaging. Organs were imaged using an IVIS Spectrum (PerkinElmer) with excitation/emission settings for Cy5 (excitation, 640 nm; emission, 680 nm).

### IPV-2–specific fecal IgA ELISA

Costar Polystyrene High Binding 96-well plates (no. 9018 Corning, Thermo Fisher Scientific, Waltham, MA) were coated with poliovirus type II monoclonal antibody (clone no. 24E2, sc-80633, Santa Cruz Biotechnology, Dallas, TX) diluted at 1:1500 in 100 mM carbonate-bicarbonate buffer (no. C3041, Sigma-Aldrich, St. Louis, MO) overnight at 4°C. Plates were then washed [using 1× PBS + 0.05% Tween 20 (BP337, Thermo Fisher Scientific, Waltham, MA), three washes] and blocked with blocking buffer [5% nonfat dry milk Blotto (no. sc-2325, Santa Cruz Biotechnology, Dallas, TX) in 1× PBS + 0.05% Tween 20] for 1 hour at 37°C. Next, plates were washed (using 1× PBS + 0.05% Tween 20, one wash) and incubated with Type 2 IPV at 10 DU/ml (Takeda, Cambridge, MA) in blocking buffer for 3 hours at RT. Plates were then washed (using 1× PBS + 0.05% Tween 20, three washes) and fecal samples were added and incubated overnight at 4°C. Before addition to the plates, the fecal samples were first concentrated using an Amicon 0.5-ml ultracentrifugal filter (MilliporeSigma, Burlington, MA) with a 10 kDa MW cutoff (14,000*g*, 3 min) and then diluted in blocking buffer starting at 1:4, followed by 2× serial dilutions. Next, plates were washed (using 1× PBS + 0.05% Tween 20, three washes) and incubated with detection antibody goat anti-rat IgA-horseradish peroxidase conjugate (no. ab97185, abcam, Waltham, MA) at 1:1000 dilution in blocking buffer for 75 min at 37°C. Plates were washed (using 1× PBS + 0.05% Tween 20, three washes) and were developed using 3,3′,5,5′-tetramethylbenzidine (TMB) substrate (no. T0440, Sigma-Aldrich, St. Louis, MO) for 25 min and stopped with 0.5 M sulfuric acid (no. 35354, VWR Scientific, Franklin, MA). The absorbance (450/630 nm) was measured on a Tecan plate reader (TECAN Infinite M PLEX, Tecan Trading AG, Switzerland). All the washes were performed using the Bio Tek plate washer (405LSRVS, BioTek Instruments Inc., Winooski, VT). End-point titers were calculated as the dilution that emitted an optical density exceeding 3× background (naïve rats).

### IPV-2–specific serum IgG ELISA

Costar Polystyrene High Binding 96-well plates (no. 9018 Corning, Thermo Fisher Scientific, Waltham, MA) were coated with poliovirus type II monoclonal antibody (clone no. 24E2, sc-80633, Santa Cruz Biotechnology, Dallas, TX) diluted at 1:1500 in 100 mM carbonate-bicarbonate buffer (no. C3041, Sigma-Aldrich, St. Louis, MO) overnight at 4°C. Plates were then washed (using 1× PBS + 0.05% Tween 20 (BP337, Fisher Scientific, Waltham, MA), three washes) and blocked with blocking buffer (5% nonfat dry milk Blotto (no. sc-2325, Santa Cruz Biotechnology, Dallas, TX) in 1× PBS + 0.05% Tween 20) for 1 hour at 37°C. Next, plates were washed (using 1× PBS + 0.05% Tween 20, one wash) and incubated with Sabin poliovirus type II antigen (no. DAGC692, Creative Diagnostics, Shirley, NY) diluted at 1 μg/ml in blocking buffer for 2 hours at RT. Plates were then washed (using 1× PBS + 0.05% Tween 20, three washes) and serum samples diluted in blocking buffer starting at 1:5 followed by serial dilutions were incubated for 2 hours at 37°C. Next, plates were washed (using 1× PBS + 0.05% Tween 20, five washes) and incubated with detection antibody goat anti-rat IgG–horseradish peroxidase conjugate (no. ab97090, abcam, Walthamm, MA) diluted at 1:3000 in blocking buffer for 1 hour at 37°C. Plates were washed (using 1× PBS + 0.05% Tween 20, five washes) and were developed using TMB substrate (no. T4444, Sigma-Aldrich, St. Louis, MO) for 15 min and stopped with 0.5 M sulfuric acid (no. 35354, VWR Scientific, Franklin, MA). The absorbance (450/630 nm) was measured on a Tecan plate reader (TECAN Infinite M PLEX, Tecan Trading AG, Switzerland). All the washes were performed using the Bio Tek plate washer (405LSRVS, BioTek Instruments Inc., Winooski, VT). End-point titers were calculated as the dilution that emitted an optical density exceeding the mean + (3× SD) of naïve rats.

### Poliovirus neutralizing assay

A standard microneutralization assay targeting poliovirus type 2 was performed as described previously (*51*). In summary, serum samples were serially diluted and then incubated with poliovirus type 2 at 35°C for 3 hours. The mixtures were subsequently applied to HEp-2(C) cells and incubated at 35°C for 5 days. Postincubation, cells were stained with crystal violet, and cell viability was assessed by measuring optical density at 595 nm. Titers were calculated using the Spearman-Karber method. Seropositivity was defined as antibody titers of 1:8 (3.00 log2) or higher, with the range of antibody titers spanning from 2.50 to 10.50 log2.

This activity underwent review by the Centers for Disease Control and Prevention (CDC) and was conducted in accordance with applicable federal laws and CDC policies (refer to 45 C.F.R. part 46, 21 C.F.R. part 56; 42 U.S.C. §241(d); 5 U.S.C. §552a; 44 U.S.C. §3501 et seq.).

### Histology and IHC staining of rat small intestine

Immediately after harvesting, 0.5-cm segments of the distal ileum (10 cm from the ileocecal junction) were flushed with PBS and fixed in 4% PFA in PBS for 24 hours. The samples were then transferred to 70% ethanol for further analysis. Fixed tissues were processed in paraffin (Tek VIP 5 tissue processor, Sakura Finetek, Torrence, CA, USA), embedded, and sectioned at 5 μm using a microtome (Thermo Shandon Finesse). Sectioning was performed at 500-μm step intervals to a depth of approximately 2500 μm, or until three cross sections were obtained.

For IHC staining, sections were deparaffinized, rehydrated, and placed in a pressure cooker at 123°C for 5 min, (DeCloaking chamber BioCare Medical Pacheco, CA) with tris-EDTA pH 9.0 antigen retrieval buffer. After antigen retrieval, slides were quenched using 0.03% hydrogen peroxide (no. ab64218 Abcam, Waltham MA) for 10 min and blocked with Animal Free blocking solution (no. 15019 Cell Signaling Technologies, Danvers, MA) for 30 min at RT. After rinsing the slides, they were incubated with primary antibody anti-IgA mouse (monoclonal (clone SPM217), no. ab212330, Abcam, Waltham, MA) at 1:500 and E-cadherin rabbit (polyclonal, no. A11492, Abclonal Technology, Woburn, MA) at 1:100 in 5% BSA in PBS for 90 min at RT. After washing with PBS-T, slides were incubated with Alexa Fluor 488 (goat anti-rabbit, polyclonal, no. ab150077, Abcam, Cambridge, UK) and Alexa Fluor 647 (goat anti-mouse, polyclonal, no. ab150115, Abcam, Cambridge, UK) conjugated secondary antibody for 30 min at RT. After another wash, slides were counterstained with DAPI (no. 62248 Thermo Fisher Scientific, Waltham, MA) at 1:1000 in PBS. (LabVision IHC AutoStainer, Thermo Fisher, Waltham, MA). Next, tissues were mounted with ProLong Gold Antifade (P36984, Thermo Fisher Scientific, Waltham, MA) and stored at 4°C until further confocal imaging.

### Confocal imaging and data processing

Olympus FV1200 scanning confocal microscope was used to image the immunostained intestine samples. Lasers with excitation wavelengths of (405, 473, and 635 nm) and a 30× (oil) objective were used. Z-stack images were collected at 2-μm steps. The recorded images were processed by ImageJ to quantify the IgA-containing area. For each field, the number of nuclei was determined from the DAPI channel, and the IgA-positive area was normalized to the number of nuclei.

### Multiplex analysis of cytokines in serum samples

This study used Luminex xMAP technology for multiplexed quantification of 27 Rat cytokines, chemokines, and growth factors. The multiplexing analysis was performed using the Luminex 200 system (Luminex, Austin, TX, USA) by Eve Technologies Corp. (Calgary, Alberta). Twenty-seven markers were simultaneously measured in the samples using Eve Technologies’ Rat Cytokine 27-Plex Discovery Assay (MilliporeSigma, Burlington, Massachusetts, USA) according to the manufacturer’s protocol. The 27-plex consisted of epidermal growth factor, eotaxin, fractalkine, granulocyte colony-stimulating factor, granulocyte-macrophage colony-stimulating factor, GRO/KC, IFN-γ, IL-1α, IL-1β, IL-2, IL-4, IL-5, IL-6, IL-10, IL-12 (p70), IL-13, IL-17A, IL-18, IP-10, leptin, LIX (lipopolysaccharide-induced CXC chemokine), MCP-1 (monocyte chemoattractant protein-1/CCL2), MIP-1α (macrophage inflammatory protein-1 alpha), MIP-2, RANTES, TNF-α, and vascular endothelial growth factor. Assay sensitivities of these markers range from 0.3 to 30.7 pg/ml for the 27-plex. Individual analyte sensitivity values are available in the MilliporeSigma MILLIPLEX MAP protocol.

### Spleen harvest and single-cell isolation

Spleens were harvested at week-10 postimmunization and processed into single-cell suspensions using the Spleen Dissociation Kit (Miltenyi Biotec, no. 130-095-926) according to the manufacturer’s protocol. Red blood cells were lysed using Red Blood Cell Lysis Solution (Miltenyi Biotec, no. 130-094-183) following the manufacturer’s instructions. Following isolation, cells were counted and cryopreserved in freezing medium containing DMSO, FBS, and RPMI, and stored at −80°C until further analysis.

### Intracellular cytokine staining

Polyfunctional CD4^+^ and CD8^+^ T cell responses from rat splenocytes were quantified using polio antigen-stimulated intracellular cytokine staining assays. A total of 2 × 10^6^ cells were plated and stimulated with Sabin poliovirus type II antigen (no. DAGC692, Creative Diagnostics, Shirley, NY) (2 μg/ml) or DMSO control for 1 hour, followed by GolgiStop/GolgiPlug (BD Biosciences) overlay for 6 hours at 37°C. Splenocytes were then stained with a Fixable Viability Near-IR dye (Invitrogen), followed by surface cell staining with CD8a-BUV395 (clone OX-8, BD Biosciences), CD8b-BV650 (clone 341, BD Biosciences), CD4-BUV496 (clone OX-38, BD Biosciences), CD45R/B220-BUV737 (clone HIS24, BD Biosciences), TCRγ/δ-BV510 (clone V65, BD Biosciences), CD62L-BV711 (clone HRL-1, BD Biosciences), XCR1-BV785 (clone ZET, BioLegend), CD45-PE/Dazzle 594 (clone OX-1, BioLegend), CD44-BB700 (clone OX-49, BD Biosciences), CD161/NKR-P1-PE-Cy7 (clone 3.2.3, BioLegend), and MHC-II-APC (clone HIS19, Thermo Fisher Scientific) in magnetic-activated cell sorting solution (Miltenyi Biotec) supplemented with 2% BSA (Miltenyi Biotec) and 1.5% penicillin-streptomycin (Thermo Fisher Scientific) before permeabilization with Cytofix/Cytoperm (BD Biosciences) and staining intracellularly with CD3-BV421 (clone 1F4, BD Biosciences), CD68–Alexa Fluor 488 (clone ED1, BioRad), and IFN-γ–PE (clone DB-1, BD Biosciences) in Perm/Wash (BD Biosciences). All antibodies were used at 1:100 dilution. Cells were then fixed in 2% formaldehyde and stored at 4°C until flow cytometry on an LSR II flow cytometer (BD Biosciences).

### ELISPOT

ELISPOT assays were performed using the Rat IFN-γ ELISpot kit (R&D Systems, catalog no. EL585) according to the manufacturer’s instructions. Splenocytes were plated at a density of 5 × 10^5^ cells per well and stimulated with Sabin poliovirus type II antigen (no. DAGC692, Creative Diagnostics, Shirley, NY) (1 μg per well) or left unstimulated as a control. Following incubation, plates were processed and developed according to the manufacturer’s protocol. Once plates were fully dried, spots were enumerated using an ELISPOT reader system. Antigen-specific responses were calculated by subtracting background signal from unstimulated wells.

### Statistical analysis

All data are presented as mean ± SD or mean ± SEM, data presentation and sample size for statistical analysis of individual experiments are specified in the figure captions. Statistical significance of data was determined using one-way analysis of variance (ANOVA) with a subsequent Tukey post hoc test as implemented in GraphPad Prism software. *P* values are represented by: *P ≤ 0.05; **P ≤ 0.01; ***P ≤ 0.001; ****P ≤ 0.0001.

## Supplementary Materials

### This PDF file includes:

Figs. S1 to S19 Table S1 List of competing interests
