# Supplementary material for: Am80-lipid nanoparticles serve as an enteric mucosal adjuvant following parenteral immunization with inactivated polio vaccine
Source: Sci Adv. 2026 Jun 3;12(23):eaea5433. doi: 10.1126/sciadv.aea5433 (PMC13232564; doi:10.1126/sciadv.aea5433)
Supplement: Supplementary file 1 — Figs. S1 to S19 Table S1 List of competing interests [file sciadv.aea5433_sm.pdf]

Science Advances
AAAS

Supplementary Materials for

Am80-lipid nanoparticles serve as an enteric mucosal adjuvant following parenteral immunization with inactivated polio vaccine

Behnaz Eshaghi *et al.*

Corresponding author: Robert Langer, rlanger@mit.edu; Ana Jaklenec, jaklenec@mit.edu

Sci. Adv. **12**, eaea5433 (2026)
DOI: 10.1126/sciadv.aea5433

This PDF file includes:

Figs. S1 to S19
Table S1
List of competing interests

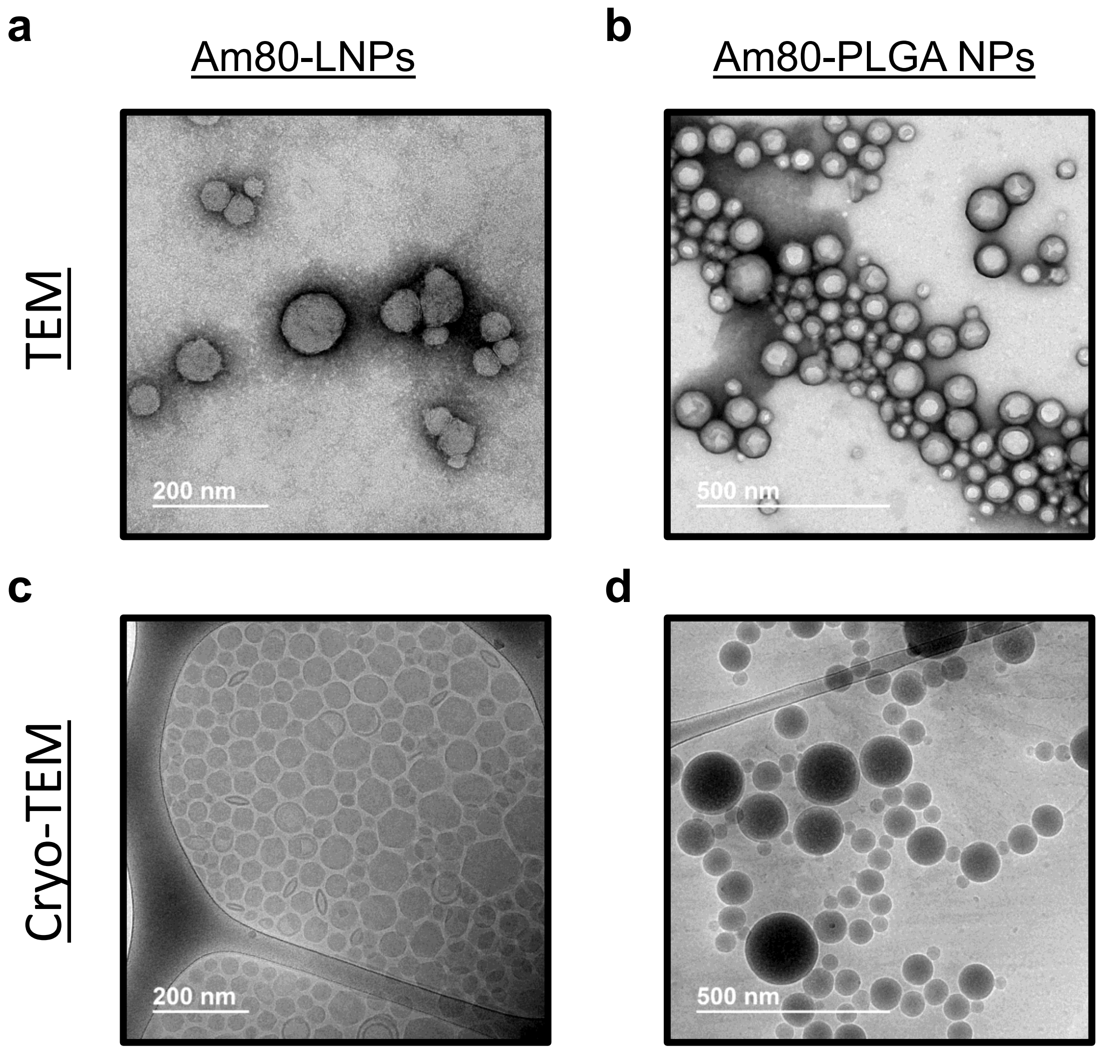

Fig. S1 High-resolution TEM and cryo-TEM images of Am80-LNPs and Am80-PLGA NPs. TEM images of (**a**) Am80-LNPs, (**b**) Am80-PLGA NPs treated with 1% (w/v) sodium phosphotungstate. Any "clustering" observed in the TEM images is a by-product of the sample preparation on the TEM grids. Cryo-TEM images of (**c**) Am80-LNPs, (**d**) Am80-PLGA NPs.

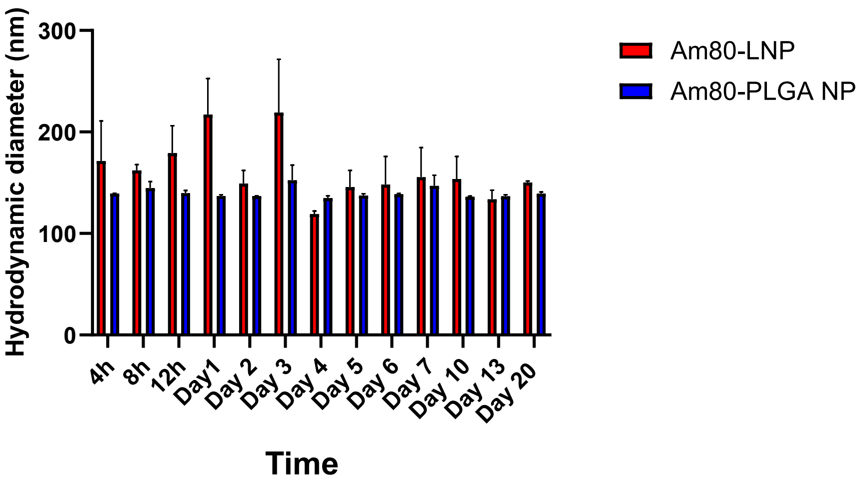

Fig. S2 Size of Am80-NPs stored at 37°C in PBS. Hydrodynamic size of the Am80-LNPs and Am80-PLGA NPs monitored during release kinetics studies at 37°C in 1× PBS at different time points.

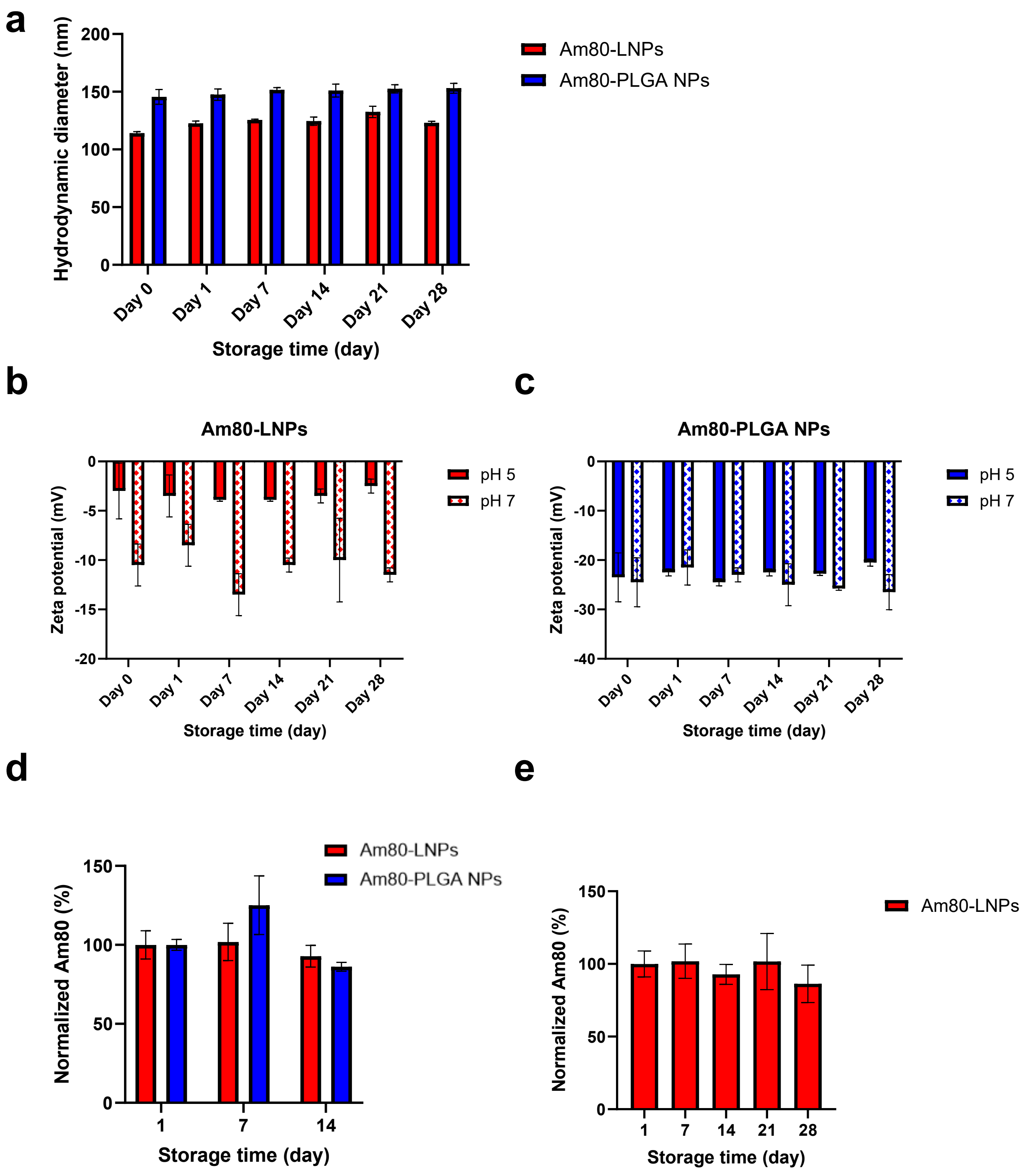

Fig. S3 Am80-NP characterization during storage at 4°C. **a)** The hydrodynamic size of Am80-LNPs and Am80-PLGA NPs at different time points. **b-c)** The zeta potential of Am80-LNPs and Am80-PLGA NPs at different time points. **d-e)** Am80 loading in Am80-LNPs and Am80-PLGA NPs during storage. Error bars represent standard deviation of three replicates.

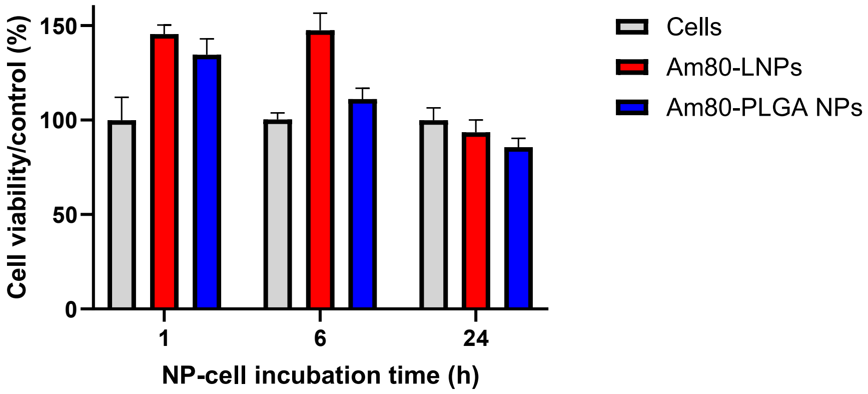

Fig. S4 MTT cell viability assay for Am80-NPs in HeLa cells. Cell viability was measured after 1, 6, and 24 h of incubation with NPs at 150 nM Am80 concentration. Cells without any treatment were included as a control.

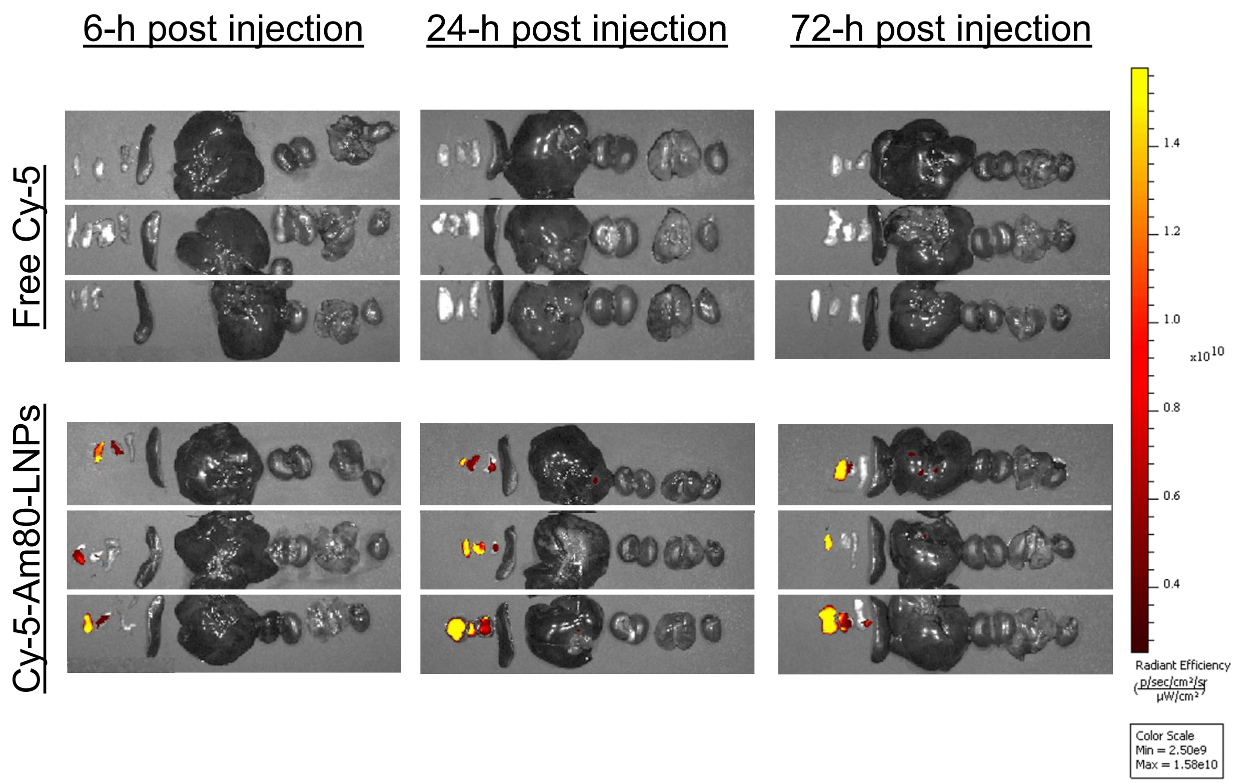

Fig. S5 Biodistribution of Cy5–Am80-LNPs following subcutaneous administration in Wistar rats. Ex vivo IVIS imaging of major organs at 6, 24, and 72 h after subcutaneous injection of PBS, free Cy5, or Cy5–Am80-LNPs. Organs are shown left to right as lymph nodes, spleen, liver, kidneys, lungs, and heart. For lymph nodes, samples were harvested and presented in the order inguinal, popliteal, and iliac. The same radiant-efficiency scale was applied to all images. Four animals were included at each time point.

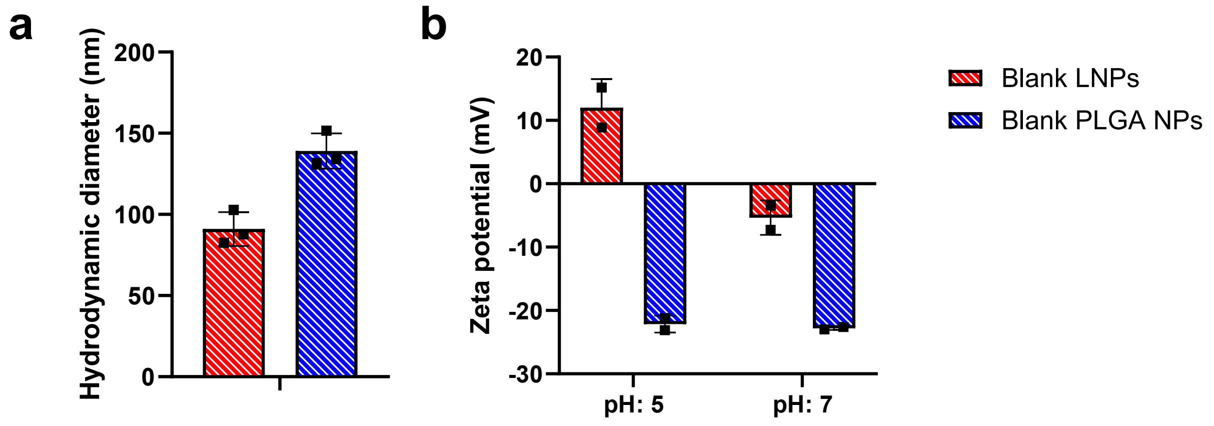

Fig. S6 Blank NP characterization. **a)** Hydrodynamic size of blank LNPs and PLGA NPs. **b)** Zeta potential of blank LNPs and PLGA NPs.

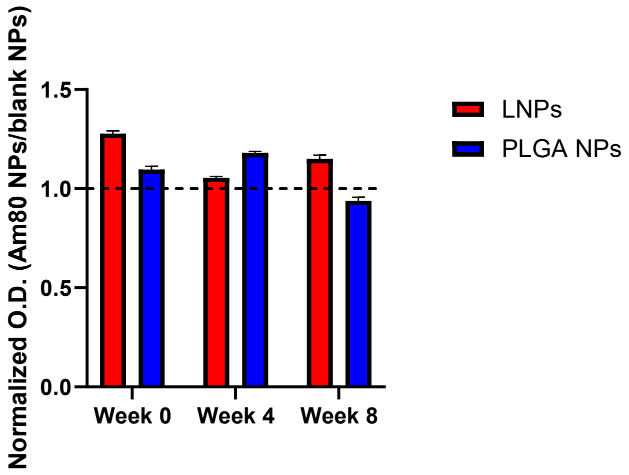

Fig. S7 Dose determination for blank NPs. Normalized absorbance values of blank NPs relative to those of Am80-NPs. The absorbance of both Am80-NPs and blank NPs was measured at 370, 375, 380, 385, and 390 nm. The dose of Am80-NPs was determined by HPLC analysis.

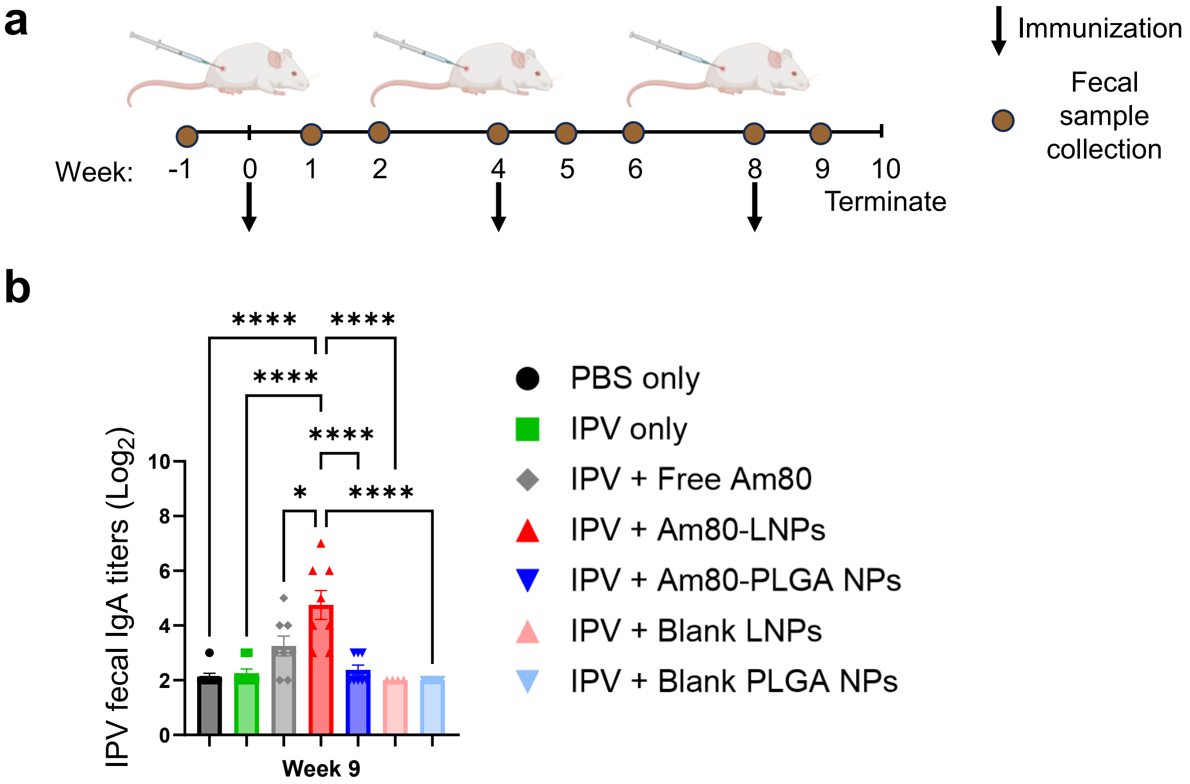

Fig. S8 IPV-2-specific fecal IgA. **a)** Schematic overview of the experimental design. Wistar rats were subcutaneously immunized at weeks 0, 4, and 8 with 12.5 DU of sIPV serotype 2 and 1.8 mg of Am80, either in free form or encapsulated in LNPs or PLGA NPs. Free Am80 was administered on days 1-3 at a dose of 0.6 mg per day. Blank LNPs and PLGA NPs were included as controls. Arrows indicate immunization, and the brown circle represents the frequency of fecal sample collection. **b)** IgA titers for individual animals at week 9. Error bars represent standard error of the mean (SEM). Statistical p-values were determined using one-way ANOVA followed by a Tukey post-hoc test. Significance levels are indicated as $*p \leq 0.05$, $**p \leq 0.01$, $***p \leq 0.001$, and $****p \leq 0.0001$. Non-significant comparisons are not shown in the figure.

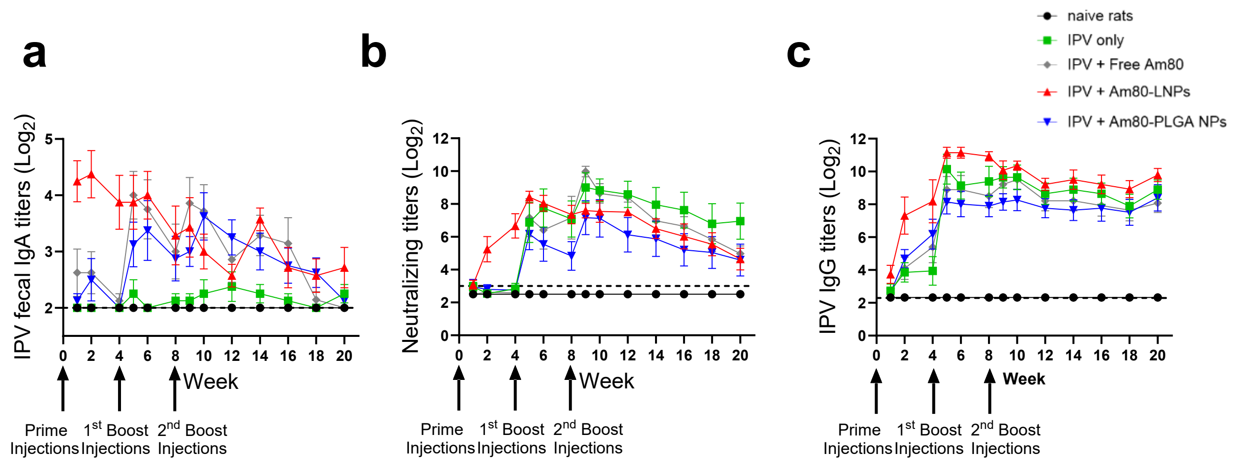

Fig. S9 Long-term evaluation of mucosal and systemic immune responses following IPV-2 vaccination with Am80 formulations. a) IPV-2-specific IgA titers in fecal samples throughout the study, arrows indicate immunization. The dashed line indicates a log2 value of 2, representing the lower limit of detection. **b)** Neutralizing titers in serum samples throughout the study, arrows indicate immunization. The dashed line indicates a log2 value of 3, representing seropositivity and protection against polio**. c)** IgG titers in serum samples throughout the study, arrows indicate the immunization. The dashed line indicates a log2 value of 2.1, representing the lower limit of detection.

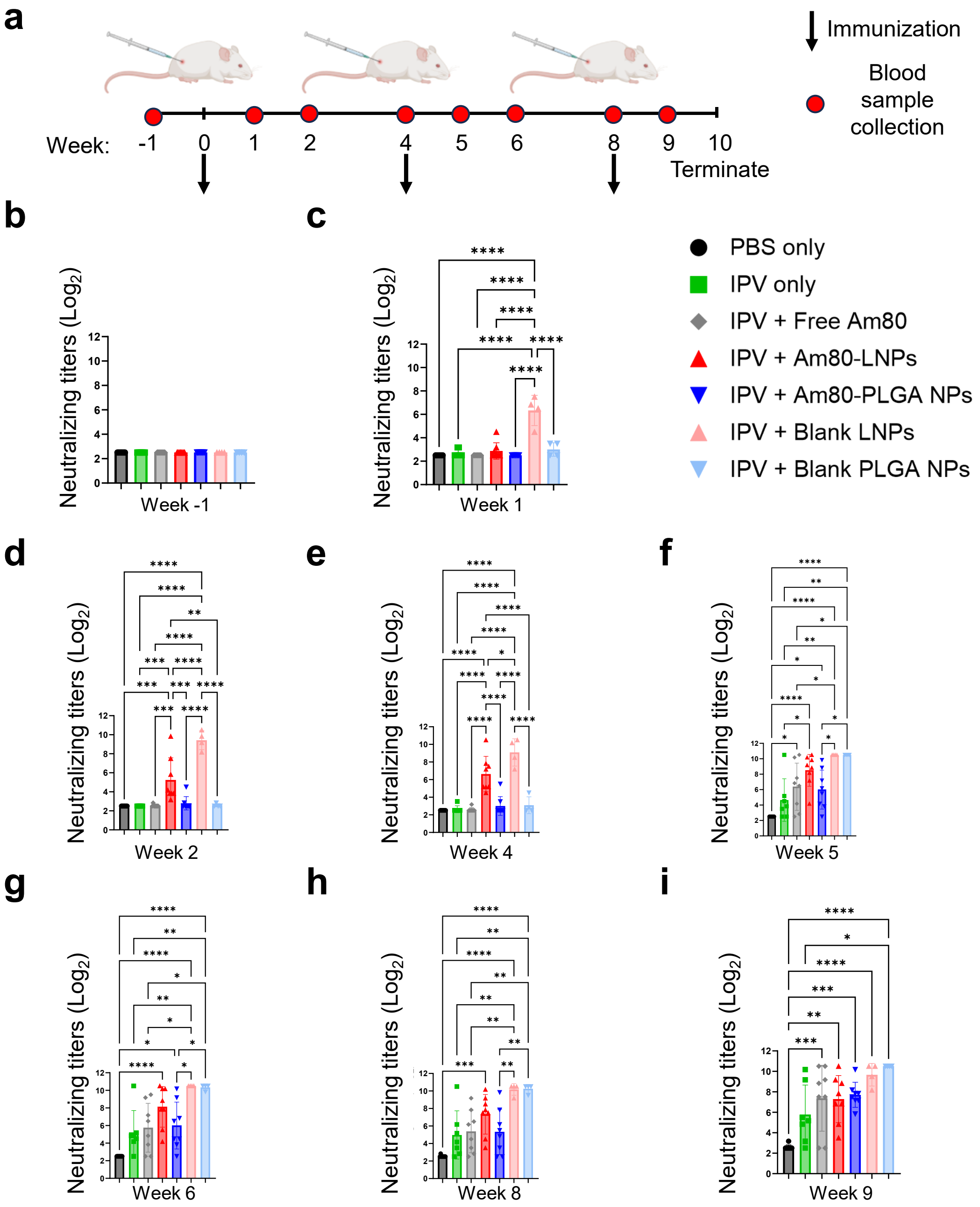

Fig. S10 Neutralizing titers in serum samples throughout the study. **a)** Schematic overview of the experimental design. Wistar rats were subcutaneously immunized at weeks 0, 4, and 8 with 12.5 DU of sIPV serotype 2 and 1.8 mg of Am80, either in free form or encapsulated in LNPs or PLGA NPs. Free Am80 was administered on days 1-3 at a dose of 0.6 mg per day. Blank LNPs and PLGA NPs were included as controls. Arrows indicate immunization, and the red circle represents the frequency of blood sample collection. **b-i)** Neutralizing titers for individual animals at different time points are shown. Error bars represent standard deviation. Statistical p-values were determined using one-way ANOVA followed by a Tukey post-hoc test. Significance levels are indicated as $*p \leq 0.05$, $**p \leq 0.01$, $***p \leq 0.001$, and $****p \leq 0.0001$. Non-significant comparisons are not shown.

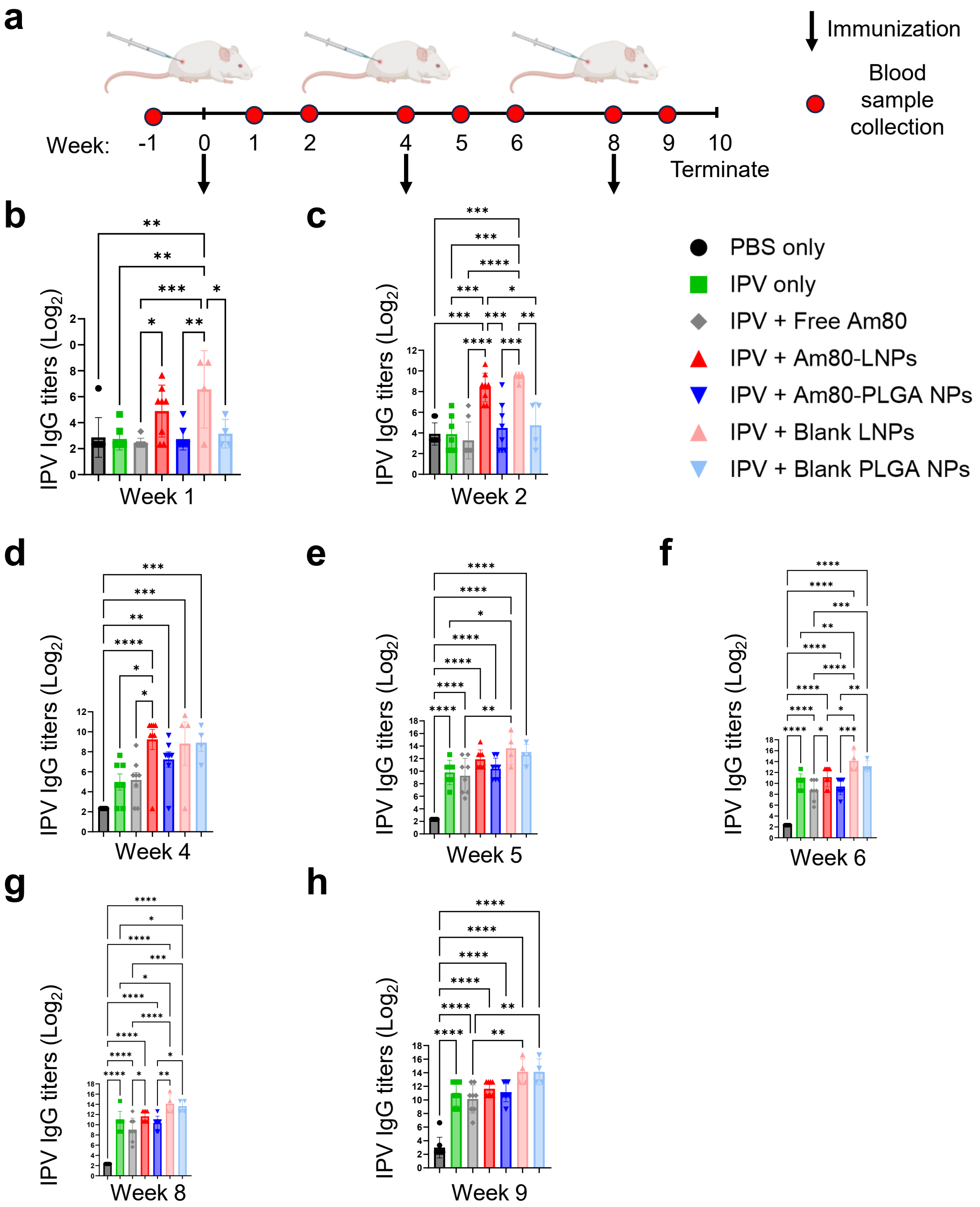

Fig. S11 IPV-2-specific IgG titers in serum samples throughout the study. **a**) Schematic overview of the experimental design. Wistar rats were subcutaneously immunized at weeks 0, 4, and 8 with 12.5 DU of sIPV serotype 2 and 1.8 mg of Am80, either in free form or encapsulated in LNPs or PLGA NPs. Free Am80 was administered on days 1-3 at a dose of 0.6 mg per day. Blank LNPs and PLGA NPs were included as controls. Arrows indicate immunization, and the red circle represents the frequency of blood sample collection. **b-h)** IPV-2-specific IgG titers for individual animals at different time points are shown. Error bars represent standard deviation. Statistical p-values were determined using one-way ANOVA followed by a Tukey post-hoc test. Significance levels are indicated as *$p \leq 0.05$, **$p \leq 0.01$, ***$p \leq 0.001$, and ****$p \leq 0.0001$. Non-significant comparisons are not shown.

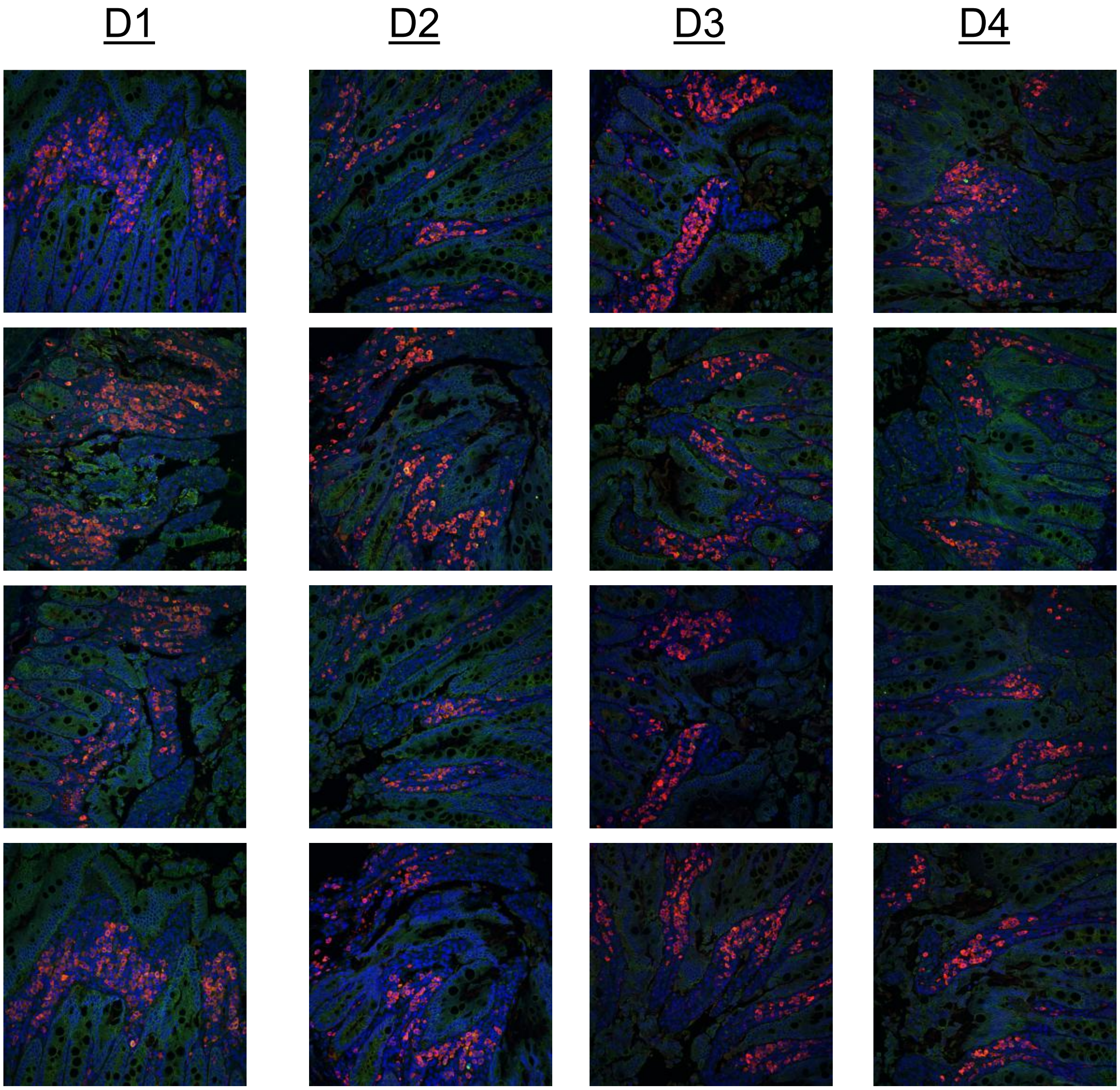

Fig. S12 IgA immunostaining of the small intestine in rats vaccinated with Am80-LNPs. Confocal *z*-stack representative images of small intestine tissues stained for IgA. Blue indicates cells, green indicates epithelial cells, and red indicates IgA. Scale bar = 50 μm.

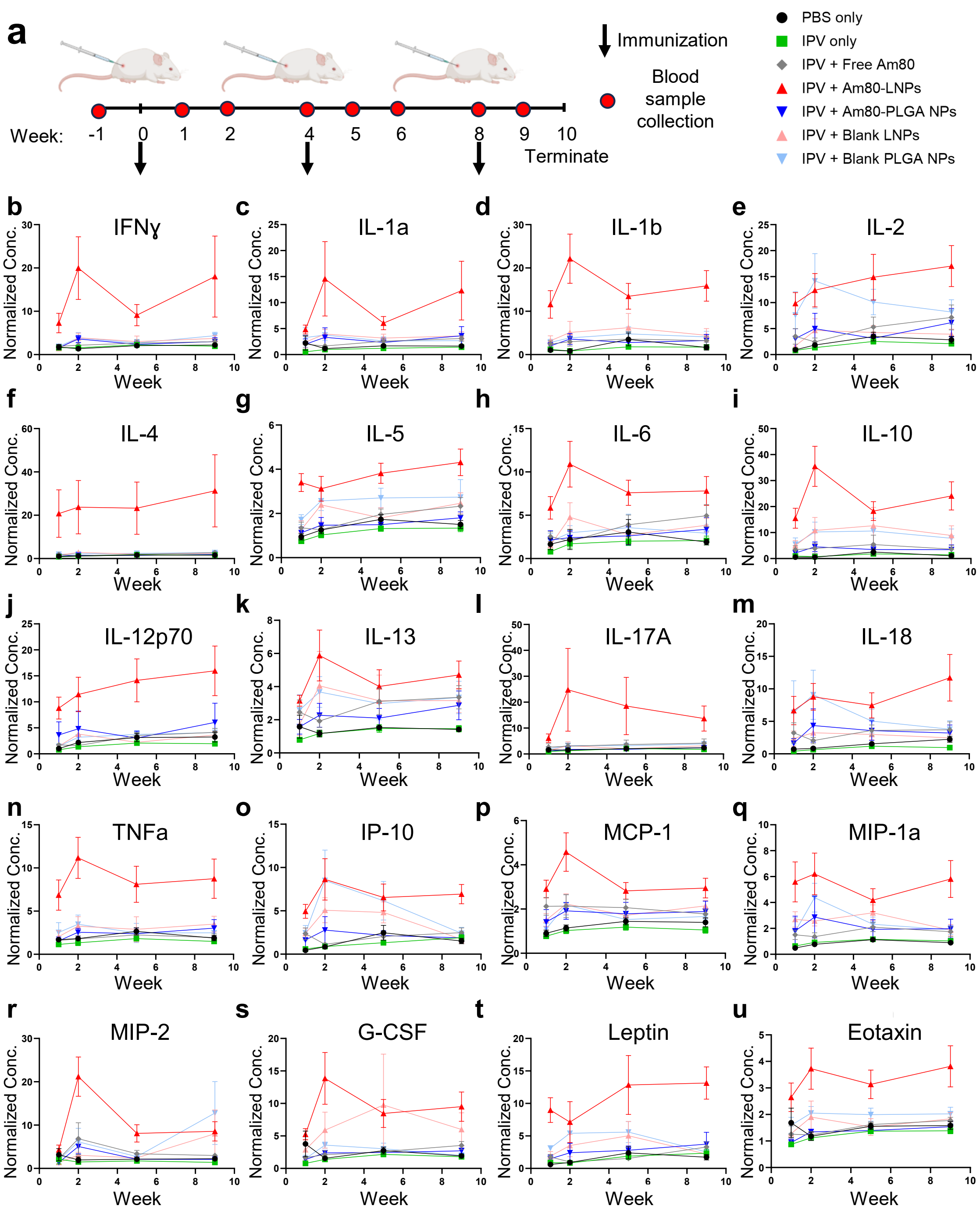

Fig. S13 Multiplexed quantification of rat cytokines, chemokines, and growth factors in serum samples collected at weeks 1, 2, 5, and 9 post-vaccination. **a)** Schematic overview of the experimental design. Wistar rats were subcutaneously immunized at weeks 0, 4, and 8 with 12.5 DU of sIPV serotype 2 and 1.8 mg of Am80, either in free form or encapsulated in LNPs or PLGA NPs. Free Am80 was administered on days 1-3 at a dose of 0.6 mg per day. Blank LNPs and PLGA NPs were included as controls. Arrows indicate immunization, and the red circle represents the frequency of blood sample collection. **b-u)** individual biomarker responses at weeks 1, 2, 5, and 9.

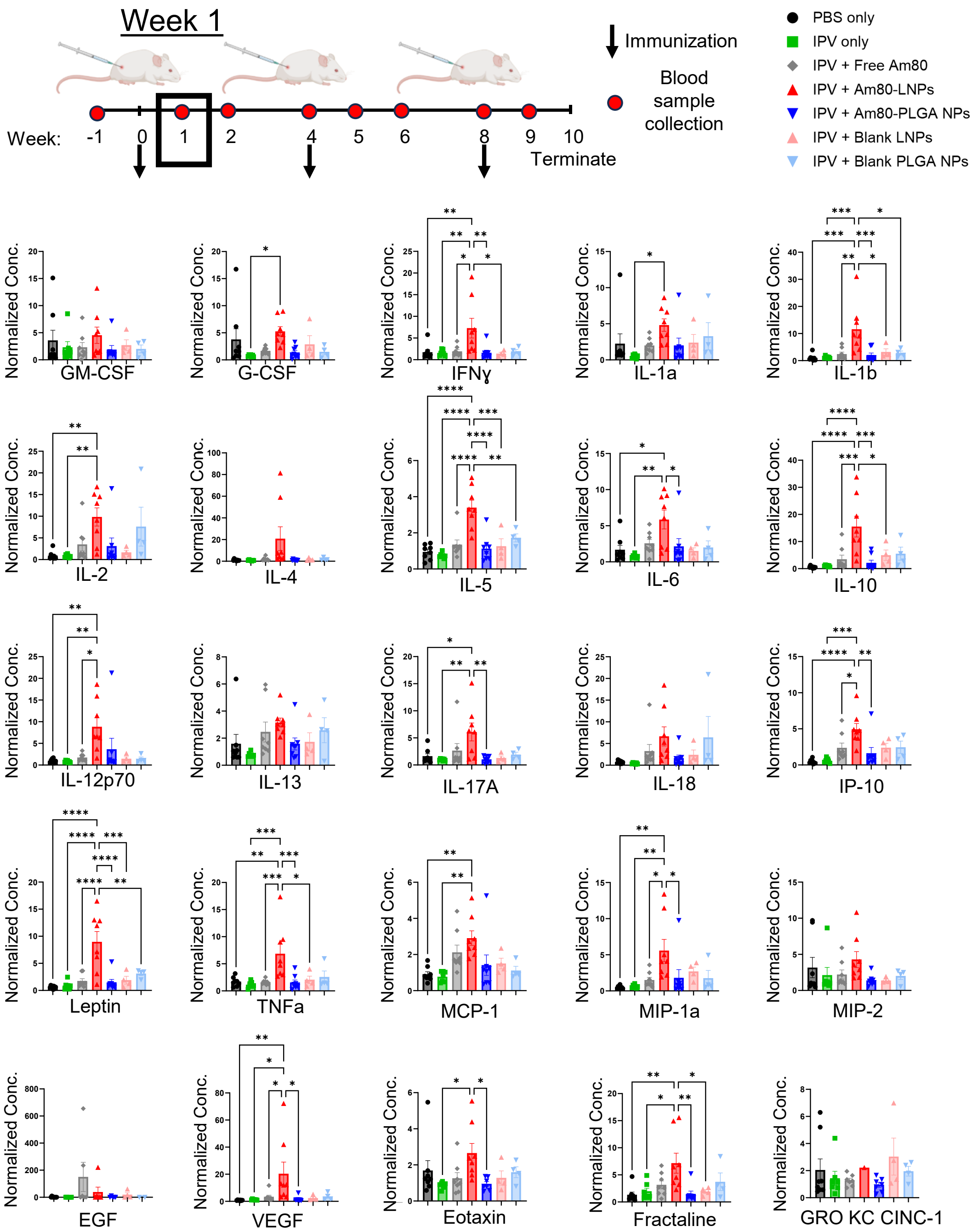

Fig. S14 Cytokine and chemokine measurements in serum samples at week 1. Schematic overview of the experimental design. Wistar rats were subcutaneously immunized at weeks 0, 4, and 8 with 12.5 DU of sIPV serotype 2 and 1.8 mg of Am80, either in free form or encapsulated in LNPs or PLGA NPs. Free Am80 was administered on days 1-3 at a dose of 0.6 mg per day. Blank LNPs and PLGA NPs were included as controls. Arrows indicate immunization, and the red circle represents the frequency of blood sample collection. All data are normalized to the baseline serum levels of each naïve rat. Error bars represent standard error of the mean (SEM). Statistical p-values were determined using one-way ANOVA followed by a Tukey post-hoc test. Significance levels are indicated as $*p \leq 0.05$, $**p \leq 0.01$, $***p \leq 0.001$, and $****p \leq 0.0001$. Non-significant comparisons are not shown.

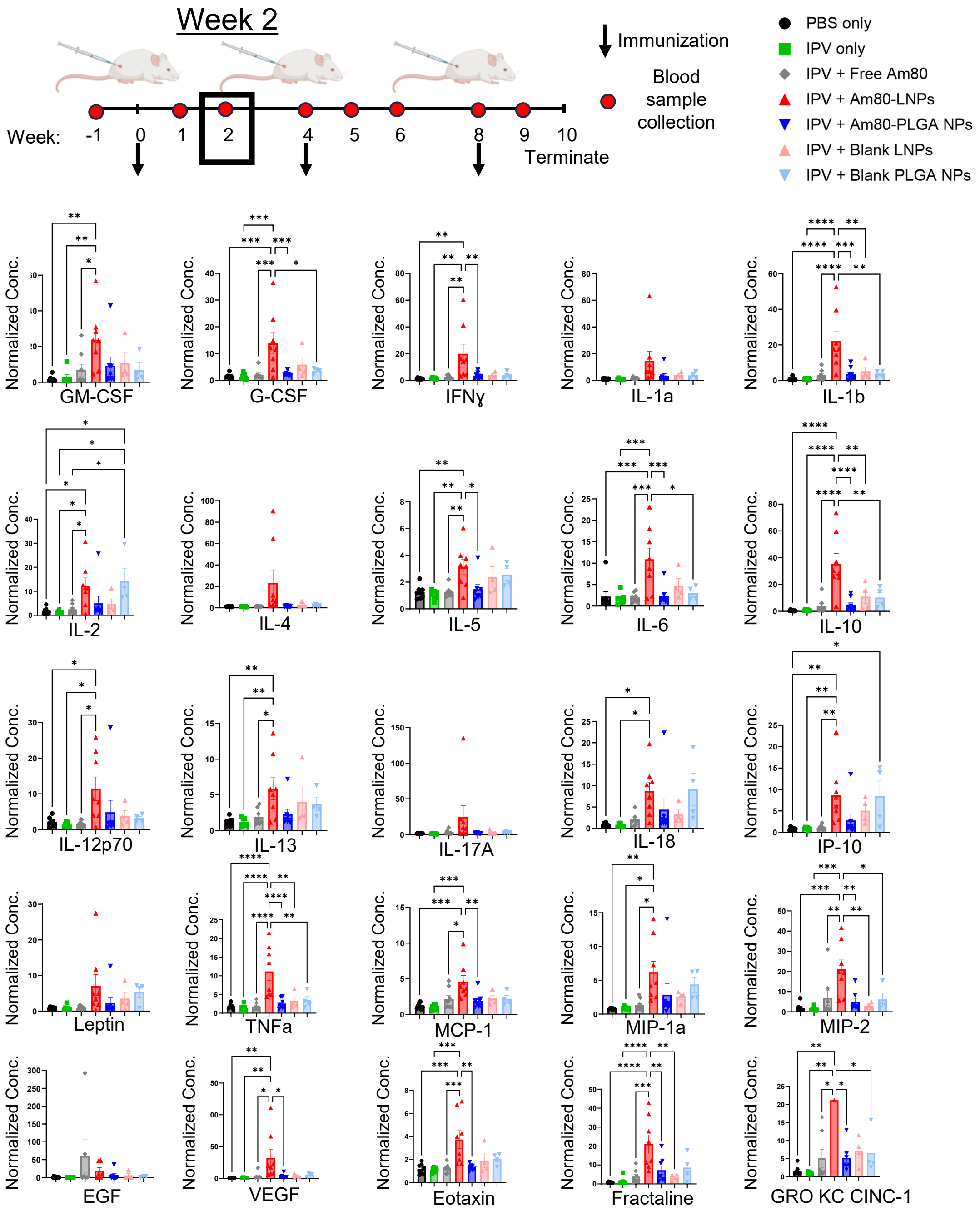

Fig. S15 Cytokine and chemokine measurements in serum samples at week 2. Schematic overview of the experimental design. Wistar rats were subcutaneously immunized at weeks 0, 4, and 8 with 12.5 DU of sIPV serotype 2 and 1.8 mg of Am80, either in free form or encapsulated in LNPs or PLGA NPs. Free Am80 was administered on days 1-3 at a dose of 0.6 mg per day. Blank LNPs and PLGA NPs were included as controls. Arrows indicate immunization, and the red circle represents the frequency of blood sample collection. All data are normalized to the baseline serum levels of each naïve rat. Error bars represent standard error of the mean (SEM). Statistical p-values were determined using one-way ANOVA followed by a Tukey post-hoc test. Significance levels are indicated as *$p \leq 0.05$, **$p \leq 0.01$, ***$p \leq 0.001$, and ****$p \leq 0.0001$. Non-significant comparisons are not shown.

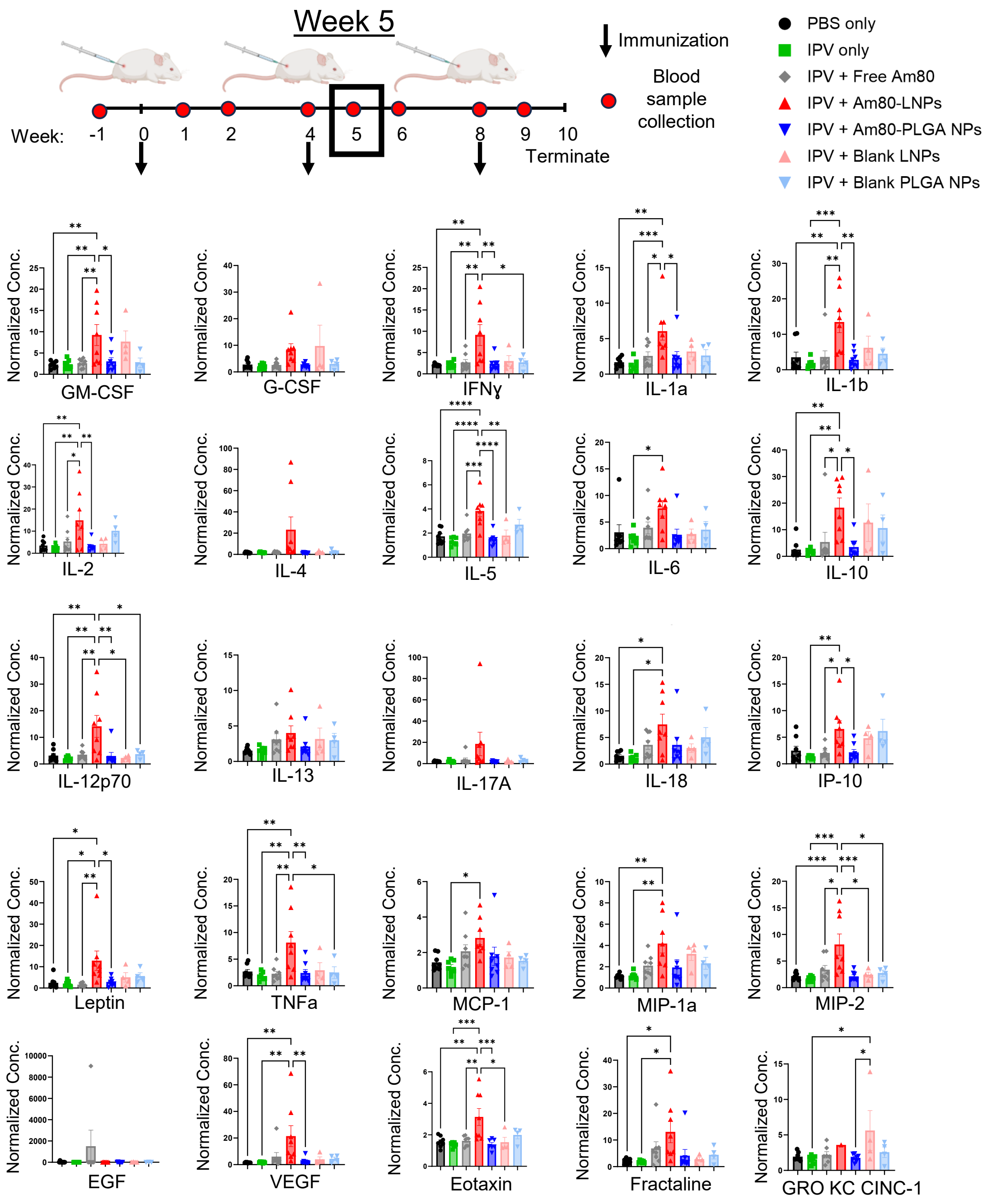

Fig. S16 Cytokine and chemokine measurements in serum samples at week 5. Schematic overview of the experimental design. Wistar rats were subcutaneously immunized at weeks 0, 4, and 8 with 12.5 DU of sIPV serotype 2 and 1.8 mg of Am80, either in free form or encapsulated in LNPs or PLGA NPs. Free Am80 was administered on days 1-3 at a dose of 0.6 mg per day. Blank LNPs and PLGA NPs were included as controls. Arrows indicate immunization, and the red circle represents the frequency of blood sample collection. All data are normalized to the baseline serum levels of each naïve rat. Error bars represent standard error of the mean (SEM). Statistical p-values were determined using one-way ANOVA followed by a Tukey post-hoc test. Significance levels are indicated as $*p \leq 0.05$, $**p \leq 0.01$, $***p \leq 0.001$, and $****p \leq 0.0001$. Non-significant comparisons are not shown.

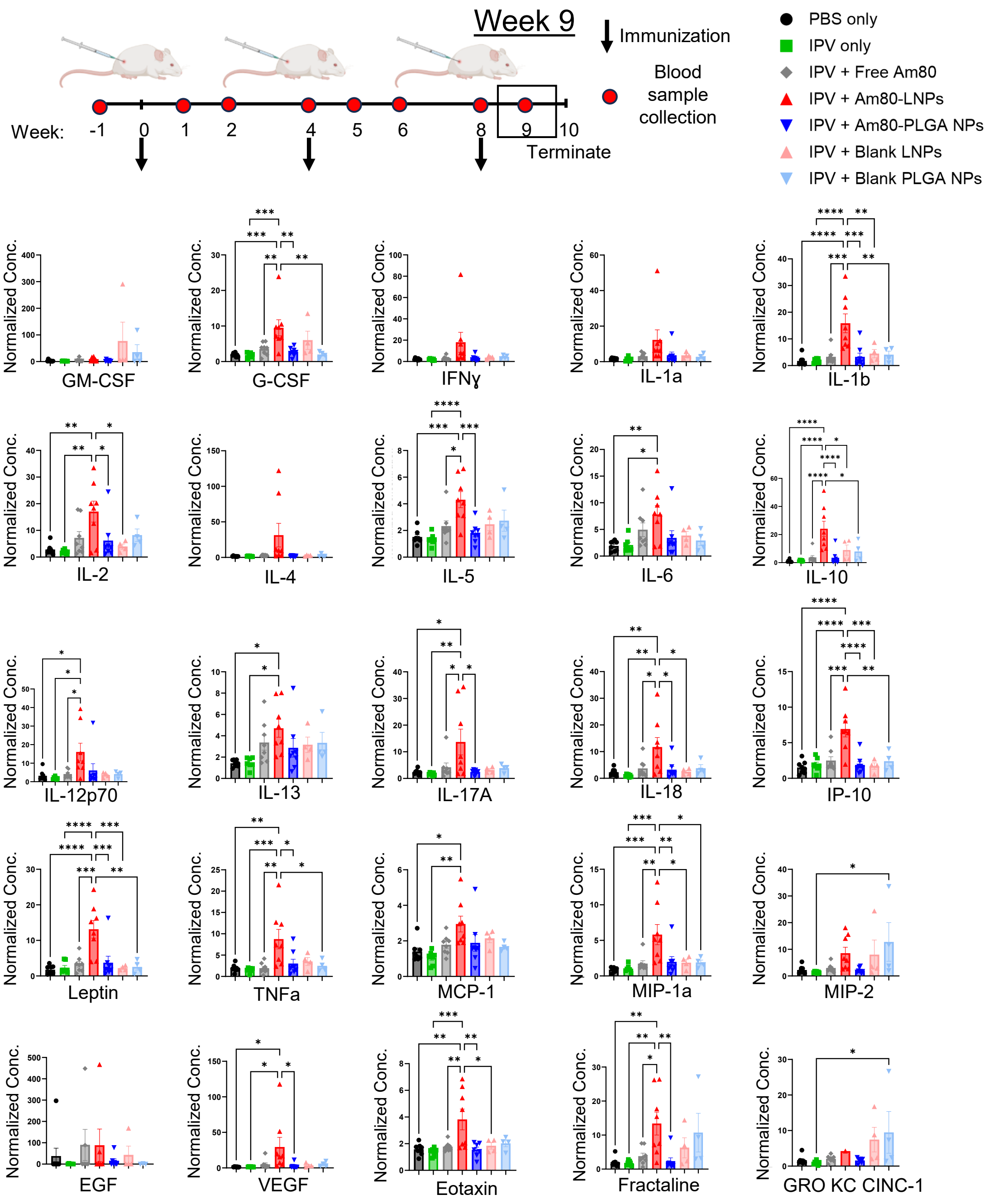

Fig. S17 Cytokine and chemokine measurements in serum samples at week 9. Schematic overview of experimental design. Wistar rats were subcutaneously immunized at weeks 0, 4, and 8 with 12.5 DU of sIPV serotype 2 and 1.8 mg of Am80, either in free form or encapsulated in LNPs or PLGA NPs. Free Am80 was administered on days 1-3 at a dose of 0.6 mg per day. Blank LNPs and PLGA NPs were included as controls. Arrows indicate immunization, and the red circle represents the frequency of blood sample collection. All data are normalized to the baseline serum levels of each naïve rat. Error bars represent standard error of the mean (SEM). Statistical p-values were determined using one-way ANOVA followed by a Tukey post-hoc test. Significance levels are indicated as *p ≤ 0.05, **p ≤ 0.01, ***p ≤ 0.001, and ****p ≤ 0.0001. Non-significant comparisons are not shown.

EGF			Eotaxin			Fractalkine			G-CSF			GM-CSF			GRO/KC/CINC-1		
FI	Obs Conc.	Exp Conc.	FI	Obs Conc.	Exp Conc.	FI	Obs Conc.	Exp Conc.	FI	Obs Conc.	Exp Conc.	FI	Obs Conc.	Exp Conc.	FI	Obs Conc.	Exp Conc.
---		0.06	---		1.22	---		0.61	---		1.22	---		3.05	---		3.66
27	0.12	0.24	7	4.99	4.88	14	2.44	2.44	6	4.88	4.88	---		12.21	16	15.35	14.65
53	1.64	0.98	26	19.24	19.53	24	9.77	9.77	13	19.53	19.53	8	48.83	48.83	23	57.54	58.59
82	3.52	3.91	354	80.00	78.13	66	39.06	39.06	33	78.12	78.13	15	195.31	195.31	71	239.00	234.38
246	15.17	15.63	2,578	307.09	312.50	200	156.25	156.25	107	312.50	312.50	39	781.25	781.25	265	883.05	937.50
863	64.20	62.50	8,675	1,193.38	1,250.00	680	625.00	625.00	506	1,250.00	1,250.00	199	3,125.00	3,125.00	1,120	4,445.96	3,750.00
2,913	249.71	250.00	17,192	6,924.17	5,000.00	1,753	2,500.00	2,500.00	1,742	5,000.00	5,000.00	809	12,500.00	12,500.00	2,446	13,153.25	15,000.00
7,363	1,000.10	1,000.00	19,430	14,482.31	20,000.00	3,719	10,000.00	10,000.00	6,185	20,000.00	20,000.00	3,077	50,000.00	50,000.00	5,947	63,948.69	60,000.00

IFNγ			IL-1α			IL-1β			IL-2			IL-4			IL-5		
FI	Obs Conc.	Exp Conc.	FI	Obs Conc.	Exp Conc.	FI	Obs Conc.	Exp Conc.	FI	Obs Conc.	Exp Conc.	FI	Obs Conc.	Exp Conc.	FI	Obs Conc.	Exp Conc.
---		3.66	---		3.05	---		0.61	---		3.05	---		1.22	---		1.22
11	14.65	14.65	6	9.93	12.21	25	OOR <	2.44	14	12.21	12.21	---		4.88	---		4.88
16	58.59	58.59	16	67.75	48.83	39	31.53	9.77	22	48.83	48.83	9	20.50	19.53	7	19.78	19.53
43	234.37	234.38	35	164.63	195.31	45	49.28	39.06	54	195.31	195.31	21	75.18	78.13	21	77.45	78.13
153	937.50	937.50	161	781.87	781.25	83	160.98	156.25	173	781.25	781.25	128	321.29	312.50	191	314.56	312.50
800	3,750.00	3,750.00	877	4,218.77	3,125.00	212	543.20	625.00	653	3,125.00	3,125.00	886	1,238.51	1,250.00	1,993	1,245.83	1,250.00
3,259	14,999.99	15,000.00	2,200	10,828.71	12,500.00	902	2,840.77	2,500.00	2,008	12,500.00	12,500.00	4,365	5,016.99	5,000.00	8,060	5,036.81	5,000.00
6,763	60,000.00	60,000.00	9,573	52,495.74	50,000.00	2,629	9,792.41	10,000.00	3,352	50,000.00	50,000.00	5,842	19,401.95	20,000.00	9,708	18,700.16	20,000.00

IL-6			IL-10			IL-12p70			IL-13			IL-17A			IL-18		
FI	Obs Conc.	Exp Conc.	FI	Obs Conc.	Exp Conc.	FI	Obs Conc.	Exp Conc.	FI	Obs Conc.	Exp Conc.	FI	Obs Conc.	Exp Conc.	FI	Obs Conc.	Exp Conc.
---		18.31	28	OOR <	1.83	---		3.05	---		1.22	---		1.83	---		3.05
---		73.24	40	18.26	7.32	---		12.21	7	OOR <	4.88	8	7.32	7.32	8	12.21	12.21
9	292.97	292.97	54	53.97	29.30	9	48.83	48.83	13	39.01	19.53	13	29.30	29.30	16	48.83	48.83
16	1,171.87	1,171.88	73	111.23	117.19	21	195.31	195.31	22	75.65	78.13	46	117.19	117.19	45	195.31	195.31
48	4,687.50	4,687.50	167	428.37	468.75	81	781.25	781.25	122	303.95	312.50	175	468.75	468.75	119	781.25	781.25
216	18,750.00	18,750.00	562	2,028.82	1,875.00	393	3,125.00	3,125.00	944	1,285.38	1,250.00	1,109	1,875.00	1,875.00	567	3,125.00	3,125.00
953	75,000.00	75,000.00	1,611	7,031.05	7,500.00	1,288	12,500.00	12,500.00	4,603	4,933.54	5,000.00	4,819	7,500.00	7,500.00	1,954	12,500.00	12,500.00
3,536	300,000.00	300,000.00	5,845	31,049.12	30,000.00	5,188	50,000.00	50,000.00	10,706	20,187.30	20,000.00	12,337	30,000.00	30,000.00	3,815	50,000.00	50,000.00

IP-10			Leptin			LIX			MCP-1			MIP-1α			MIP-2		
FI	Obs Conc.	Exp Conc.	FI	Obs Conc.	Exp Conc.	FI	Obs Conc.	Exp Conc.	FI	Obs Conc.	Exp Conc.	FI	Obs Conc.	Exp Conc.	FI	Obs Conc.	Exp Conc.
---		0.61	---		3.66	---		6.10	---		7.32	7	0.36	0.61	---		6.10
---		2.44	31	OOR <	14.65	17	20.24	24.41	---		29.30	13	2.83	2.44	8	21.90	24.41
21	9.71	9.77	41	84.91	58.59	32	99.91	97.66	5	107.27	117.19	42	9.62	9.77	22	108.37	97.66
96	39.68	39.06	62	213.38	234.38	246	384.75	390.63	17	517.46	468.75	190	35.89	39.06	96	368.51	390.63
580	151.08	156.25	198	962.03	937.50	1,653	1,580.53	1,562.50	93	1,765.82	1,875.00	942	203.90	156.25	696	1,602.65	1,562.50
2,736	675.21	625.00	679	3,795.91	3,750.00	4,765	6,785.70	6,250.00	853	7,742.18	7,500.00	1,707	552.17	625.00	3,443	6,248.39	6,250.00
5,709	2,247.88	2,500.00	2,124	14,728.80	15,000.00	6,641	17,687.36	25,000.00	3,491	29,290.19	30,000.00	2,704	1,983.86	2,500.00	7,603	24,315.71	25,000.00
8,965	11,202.73	10,000.00	5,996	60,388.12	60,000.00	8,945	282,853.67	100,000.00	4,698	147,897.09	120,000.00	3,735	19,678.37	10,000.00	9,096	116,757.68	100,000.00

RANTES			TNFα			VEGF		
FI	Obs Conc.	Exp Conc.	FI	Obs Conc.	Exp Conc.	FI	Obs Conc.	Exp Conc.
24	1.23	1.22	13	OOR <	0.61	---		1.22
81	4.73	4.88	20	4.79	2.44	---		4.88
304	20.45	19.53	28	9.54	9.77	75	18.47	19.53
982	75.12	78.13	84	39.24	39.06	176	87.43	78.13
3,631	328.46	312.50	342	149.99	156.25	434	284.80	312.50
9,958	1,187.83	1,250.00	1,607	656.21	625.00	1,572	1,334.36	1,250.00
19,052	5,935.76	5,000.00	3,852	2,346.55	2,500.00	4,033	4,871.70	5,000.00
20,686	13,526.80	20,000.00	5,063	13,207.19	10,000.00	7,364	20,268.78	20,000.00

Fig. S18 Detected concentration ranges of each biomarker. Each box displays the fluorescence intensity (first column) alongside the observed (second column) and expected (third column) concentrations for each biomarker. All concentrations are reported in pg/mL. For example, for EGF, the fluorescence intensity values of 27 to 7363 correspond to observed concentrations of 0.12 to 1,000.10 pg/mL.

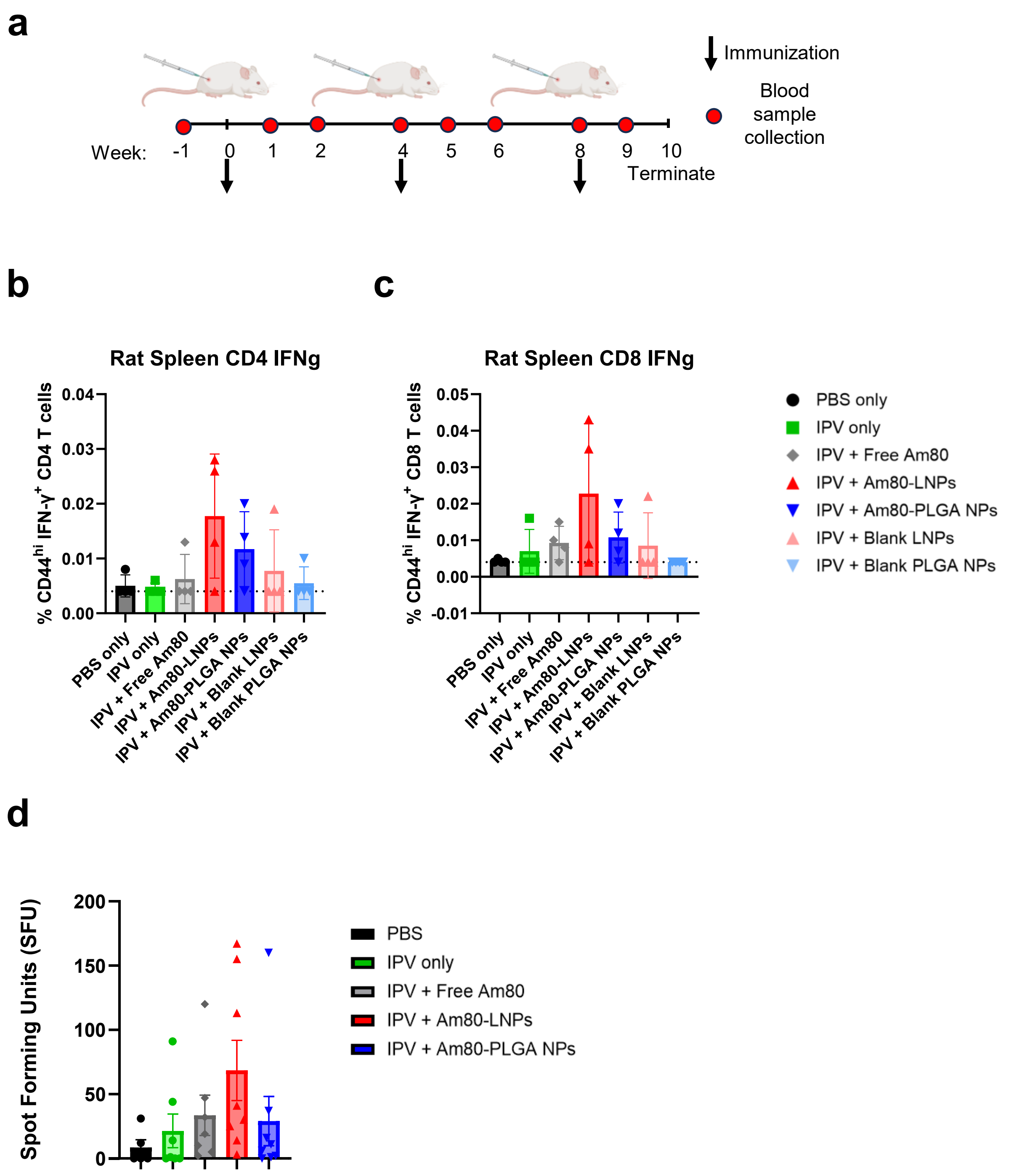

Fig. S19 Am80-LNPs enhance antigen-specific cellular immune responses following vaccination. a) Schematic overview of the immunization schedule and sample collection timeline. Spleens were harvested at week 10. **b–c)** Intracellular cytokine staining (ICS) of splenocytes showing the frequency of IFN-γ–producing $CD4^+$ (b) and $CD8^+$ (c) T cells following vaccination. **d)** ELISPOT analysis of IFN-γ–secreting splenocytes.

Table S1. Evaluation of several materials for Am80 encapsulation in NP formulations.

**Materials tested**		**Requirements**			
	**NP size (nm)**	**NP Stability**	**LN delivery / retention time**	**T-cell homing in IEL & LPL**	**Reproducible in vivo fecal IgA production**
PLGA (Including different MWs)	150-180	✓	NT	✓	NT
PLGA-PEG (Including different MWs)	150-180	✓	NT	✓	✓
Pluronic F127 polymerized with propylene sulfide (PPS)	8, 30	✓	✓	x	x
Acetalated Dextran	160	✓	✓	✓	✓
β-Cyclodextrin	2	✓	✓	NT	x
Pluronic F127	20	✓	✓	x	x

NP stability is defined as the ability to remain suspended as NPs for at least two days
IEL: Intraepithelial Lymphocytes
LPL: Lamina Propria Lymphocytes
NT: Not tested

2026 Potential Competing Interests Disclosure

From FY 2021 to the present, Dr. Robert Langer receives licensing fees (to patents in which he was an inventor on) from, invested in, consults (or was on Scientific Advisory Boards or Boards of Directors) for, lectured (and received a fee), or conducts sponsored research at MIT for which he was not paid for the following entities:

1. 611 Therapeutics
2. Abpro International
3. Alkermes
4. Alnylam Pharmaceuticals, Inc
5. aMoon
6. Aptar
7. Arsenal Medical
8. Bai Biosciences
9. BeSound;
10. Bilayer Therapeutics
11. BioInnovation Institute (Novo Nordisk Fonden)
12. Blackstone (Formerly Clarus)
13. BMG Labs;
14. Boehringer Ingelheim
15. Boston Children's Hospital
16. Cantor Fitzgerald
17. Celero
18. ClavystBio
19. Clontech Laboratories
20. Combined Therapeutics ("CTx")
21. Conference Forum
22. Coregen
23. Cybin
24. CyMon Bio
25. Daré Biosciences (Formerly Microchips Biotech, Juniper Pharmaceuticals, and Columbia Laboratories)
26. Decoy Therapeutics
27. Dewpoint Therapeutics
28. Duracyte
29. Earli
30. ELC (Esteé Lauder Companies)
31. Entrega
32. EpiBone
33. Establishment Labs, SA.
34. Everlywell
35. Evox Therapeutics, Ltd.
36. Genemedicine Co Ltd
37. Genuv
38. Genyro
39. Glycobia
40. HERVolution Therapeutics
41. HexemBio;
42. HCR (HealthCare Royalty Partners)
43. Immunai
44. InVivo Therapeutics;
45. IxBio
46. Jimini Health, Inc.
47. Johnson & Johnson
48. Kala Pharmaceuticals
49. Kendall Capital
50. Kensa
51. Klotho Neuro
52. Landsdowne Labs;
53. LikeMinds;
54. Lindus Health
55. Luminopia, Inc.
56. Lyndra Therapeutics
57. Lyra Therapeutics (Formerly 480 Biomedical)
58. Marble Therapeutics
59. Matrisome Bio;
60. McGovern Institute
61. Moderna Therapeutics

2026 Potential Competing Interests Disclosure

From FY 2021 to the present, Dr. Robert Langer receives licensing fees (to patents in which he was an inventor on) from, invested in, consults (or was on Scientific Advisory Boards or Boards of Directors) for, lectured (and received a fee), or conducts sponsored research at MIT for which he was not paid for the following entities:

62. Nanobiosym
63. Neochromosome
64. Neoteny 4 LLP
65. NextRNA
66. Noveome Biotherapeutics, Inc.
67. Novo Nordisk
68. Okinawa Institute of Science and Technology (OIST) Graduate School (Japan)
69. OmniPulse
70. OrthoBio Therapeutics
71. Ovid Therapeutics
72. Particles for Humanity
73. Placon Therapeutics
74. Polaris Partners
75. Portal Instruments
76. PrognomIQ Inc.
77. PureTech
78. Quris (Israel)
79. ReLive
80. Department of Chemical and Biological Engineering Rensselaer Polytechnic Institute
81. Replay Bio
82. Sail Biosciences (Formerly Senda)
83. Seer, Inc.
84. Sethera Therapeutics
85. Sio2
86. Soufflé Therapeutics
87. Solvandria Foundation
88. StemBioSys, Inc.
89. Syntis Bio, Inc
90. T2 Biosystems
91. Tecnológico de Monterrey (Mexico)
92. Teal Bio
93. Third Rock Ventures
94. TISSIUM (Formerly Gecko)
95. T.Rx Capital, LLC
96. Unilever (Living Proof)
97. University of Chicago (Coleman Seskind Lecture)
98. VasoRX
99. Verseau Therapeutics, Inc.
100. VitaKey
101. Vivtex Corporation
102. WearOptimo
103. World Chemical Engineering Council
104. Xenter
105. YourBio (Formerly 7th Sense Biosystems)
106. ZWI Therapeutics
